# Discovery and structural mechanism of DNA endonucleases guided by RAGATH-18-derived RNAs

**DOI:** 10.1038/s41422-024-00952-1

**Published:** 2024-04-04

**Authors:** Kuan Ren, Fengxia Zhou, Fan Zhang, Mingyu Yin, Yuwei Zhu, Shouyu Wang, Yan Chen, Tengjin Huang, Zixuan Wu, Jiale He, Anqi Zhang, Changyou Guo, Zhiwei Huang

**Affiliations:** 1 HIT Center for Life Sciences, School of Life Science and Technology, Harbin Institute of Technology, Harbin, Heilongjiang China; 2 Westlake Center for Genome Editing, Westlake Laboratory of Life Sciences and Biomedicine, School of Life Sciences, Westlake University, Hangzhou, Zhejiang China; 3 New Cornerstone Science Laboratory, Shenzhen, Guangdong China

**Keywords:** Biological techniques, Cryoelectron microscopy

## Abstract

CRISPR-Cas systems and IS200/IS605 transposon-associated TnpBs have been utilized for the development of genome editing technologies. Using bioinformatics analysis and biochemical experiments, here we present a new family of RNA-guided DNA endonucleases. Our bioinformatics analysis initially identifies the stable co-occurrence of conserved RAGATH-18-derived RNAs (reRNAs) and their upstream IS607 TnpBs with an average length of 390 amino acids. IS607 TnpBs form programmable DNases through interaction with reRNAs. We discover the robust dsDNA interference activity of IS607 TnpB systems in bacteria and human cells. Further characterization of the *Firmicutes bacteria* IS607 TnpB system (ISFba1 TnpB) reveals that its dsDNA cleavage activity is remarkably sensitive to single mismatches between the guide and target sequences in human cells. Our findings demonstrate that a length of 20 nt in the guide sequence of reRNA achieves the highest DNA cleavage activity for ISFba1 TnpB. A cryo-EM structure of the ISFba1 TnpB effector protein bound by its cognate RAGATH-18 motif-containing reRNA and a dsDNA target reveals the mechanisms underlying reRNA recognition by ISFba1 TnpB, reRNA-guided dsDNA targeting, and the sensitivity of the ISFba1 TnpB system to base mismatches between the guide and target DNA. Collectively, this study identifies the IS607 TnpB family of compact and specific RNA-guided DNases with great potential for application in gene editing.

## Introduction

Bacteria have developed a variety of defense systems against bacteriophage infection.^1,2^ Restriction modification (RM) systems recognize and cleave invading DNA sequences at specific sites.^3^ Abortive infection (Abi) systems cause cell death or metabolic arrest after infection,^4^ whereas CRISPR-Cas systems provide adaptive immunity by integrating the short segments of invader-derived DNA into a CRISPR array.^5^ Previous studies have demonstrated that genes encoding some anti-phage defense systems are significantly clustered in bacterial and archaeal genome regions of “defense islands”.^6^ For example, the genes encoding RM systems are often located near the genes encoding Abi systems and other phage resistance systems.^7^

A series of antiviral defense systems have been identified by exploring the genes enriched in proximity to known defense genes in “defense islands;”^8–11^ the mechanisms employed by these bacterial defense systems hold significant theoretical and practical implications. For instance, CRISPR-Cas9 technology derived from CRISPR-Cas systems has brought about a revolution in the field of genome editing.^12–16^ A comprehensive understanding of bacterial defense systems and the discovery of specific molecular components within them may provide new insights into overcoming the challenges in gene editing associated with delivery, off-target effects and low editing efficiency.^17,18^

Insertion sequences (ISs) are among the most widespread mobile elements;^19^ currently, over 4000 different entries of prokaryotic that have been identified.^20^ These IS elements are categorized into various families based on factors such as their encoded sequence, organization, end type, and open reading frame (ORF) characteristics.^20^ Among these families, the IS200/IS605 and IS607 transposition systems represent two prominent members.^20^ The IS200/IS605 and IS607 transposition loci usually encode two proteins: OrfA (TnpA) transposase and an accessory OrfB (TnpB) that is dispensable for transposition.^20^ The loci are bounded by left end (LE) and right end (RE), which are partially palindromic elements.^21,22^ Recent studies have shown that the TnpB protein of IS200/IS605 is a programmable endonuclease.^23,24^ TnpB is a RuvC domain-containing protein and predicted to be an ancestor of the Cas12 nucleases of CRISPR-Cas systems.^24–27^

Here, using terabase-scale genomic and metagenomic datasets, we first found that the conserved predicted RNAs Associated with Genes Associated with Twister and Hammerhead 18 (RAGATH-18) RNAs^28^ co-exist with IS607 TnpBs. Our biochemical data demonstrate that RAGATH-18-derived RNA (reRNA) interact with IS607 TnpB to form a novel programmable reRNA-guided DNase system. In total, from 10,258 predicted IS607 TnpB systems of 9172 bacterial strains, we identified 23 IS607 TnpB ribonucleoproteins (RNPs) from 22 strains of 17 species with robust plasmid cleavage activity. Nine IS607 TnpB RNPs from 9 strains of 8 species displayed DNA interference activity in human cells. We further determined the *Firmicutes bacterium* IS607 TnpB (ISFba1 TnpB)–reRNA–target DNA structure by cryo-electron microscopy (cryo-EM). The structure reveals the mechanism underlying the recognition of miniature ISFba1 TnpB protein (387 aa) by the scaffold reRNA and provides an explanation for the biochemical data showing that ISFba1 TnpB is more sensitive to mismatches between the guide and DNA substrates than the Cas9 enzyme.

## Results

### Identification of the RAGATH-18 RNA-associated protein

**Fig. 1:**
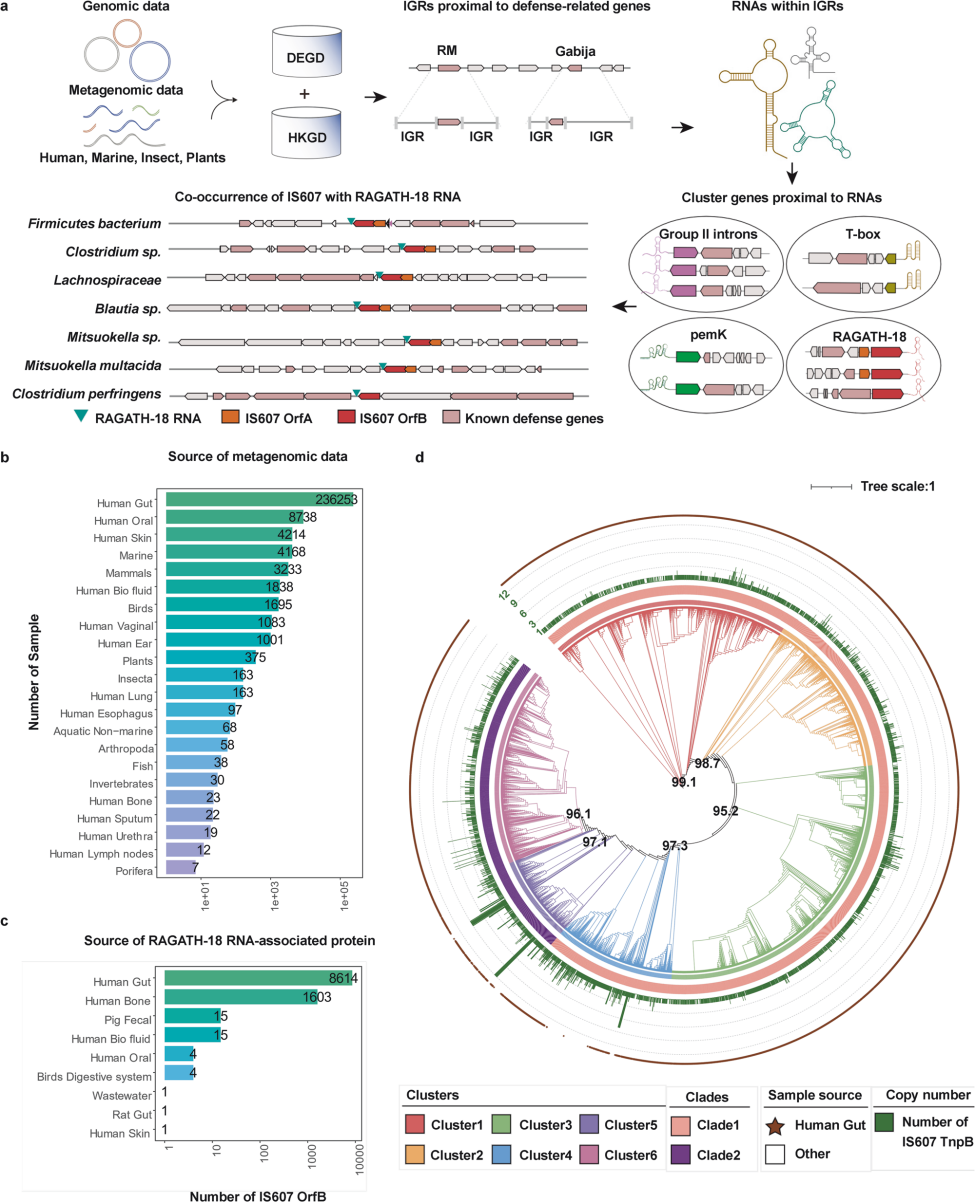
Identification of the RAGATH-18 RNA-associated protein. **a** Scheme of the computational pipeline for the identification of the RNA-associated proteins across all genomic and metagenomic data. We identified non-coding RNAs with predicted conserved secondary structures which are enriched proximal to the defense-associated genes, and clustered both five upstream and five downstream proteins of these non-coding RNAs to explore conserved cassettes. DEGD, defense associated gene database; HKGD, housekeeping gene database; RM, restriction modification; IGR, intergenic regions. Gabija is a recently described defense system.^11^ Group II introns, T-box, pemK, and RAGATH-18 are examples of predicted RNAs in the vicinity of the defense associated genes. **b** Source of metagenomic data of microbiome used for analysis. **c** Source distribution of RAGATH-18 RNA-associated protein. **d** The phylogenetic tree of RAGATH-18 RNA-associated proteins. The microbial source is shown on the outermost with the human gut microbiome in brown. The cluster identifier, clade identifier, and copy number of IS607 TnpBs are shown on the ring from innermost to the second outermost.

To discover specific molecular components in the “defense islands” of the microbial genome, we analyzed terabase-scale genomic and metagenomic data of microbiome (Fig. 1a) from a wide range of organisms including humans, other mammals, birds, fish and other marine organisms, invertebrates, insect, plants and porifera (Fig. 1b; Supplementary information, Table S1).^29–35^ We first performed a comprehensive annotation for genes related to defense systems including CRISPR-Cas, RM, and TA systems, and defined the boundaries of the “defense islands” relative to non-defense genes.^6,36,37^ Then we annotated the RNAs within intergenic regions (IGRs) proximal to defense-associated genes using Rfam^38^ (Fig. 1a). This analysis identified a number of conserved RNAs with known functions^38^ (Supplementary information, Table S1). Additionally, we discovered novel RNAs that have yet to be characterized biologically and biochemically, including RAGATH-18.^28^ To further explore whether these conserved RNAs may co-exist with any proteins, we performed gene family analysis of 5 upstream and 5 downstream proteins of the conserved RNAs (Fig. 1a). We noticed a co-occurrence of IS607 TnpBs with RAGATH-18 RNAs and 64% (10,258/16,029) of the RAGATH-18 RNAs being located next to IS607 TnpBs in 9172 strains. (Fig. 1a; Supplementary information, Table S1c). Notably, 84% (8614/10,258) of them are present in the human gut microbial metagenomic data (Fig. 1c; Supplementary information, Table S1c). The 9172 strains cover more than 130 bacterial species, which belong to seven phyla including *Firmicutes*, *Bacteroidota*, *Fusobacteria*, *Actinobacteria*, *Cyanobacteria*, *Euryarchaeota*, and *Thermotogae*. However, 97.8% of the strains are from the *Firmicutes* phylum (Supplementary information, Fig. S1a and Table S1) that is mainly comprised of low G + C Gram-positive bacteria in the human gut.^39^

We further found that 80% of the IS607 loci encode two ORFs, TnpA and TnpB, of which 30% of TnpAs are pseudo proteins due to the existence of a frameshift or an internal stop codon. By contrast, TnpB encodes a predicted RuvC domain-containing protein.^40^ Furthermore, RAGATH-18 RNAs co-occur only with IS607 TnpB but not with other IS family members. IS607 TnpBs co-existing with RAGATH-18 RNAs can be divided into two clades (six clusters) with less than 40% similarity (Fig. 1d; Supplementary information, Fig. S1b). The RAGATH-18 RNAs detected by a covariance model have an average length of 73 nucleotides (nt). The sequences among RAGATH-18 RNAs from various species had high conservation, and highly conserved nucleotides formed a possible E-loop and kink-turn.^41^ The 5’ and 3’ flanking sequences of the RAGATH-18 RNAs are conserved, approximately 50 nt and 60 nt in length, respectively.

### IS607 TnpBs are RNA-guided DNA endonucleases

**Fig. 2:**
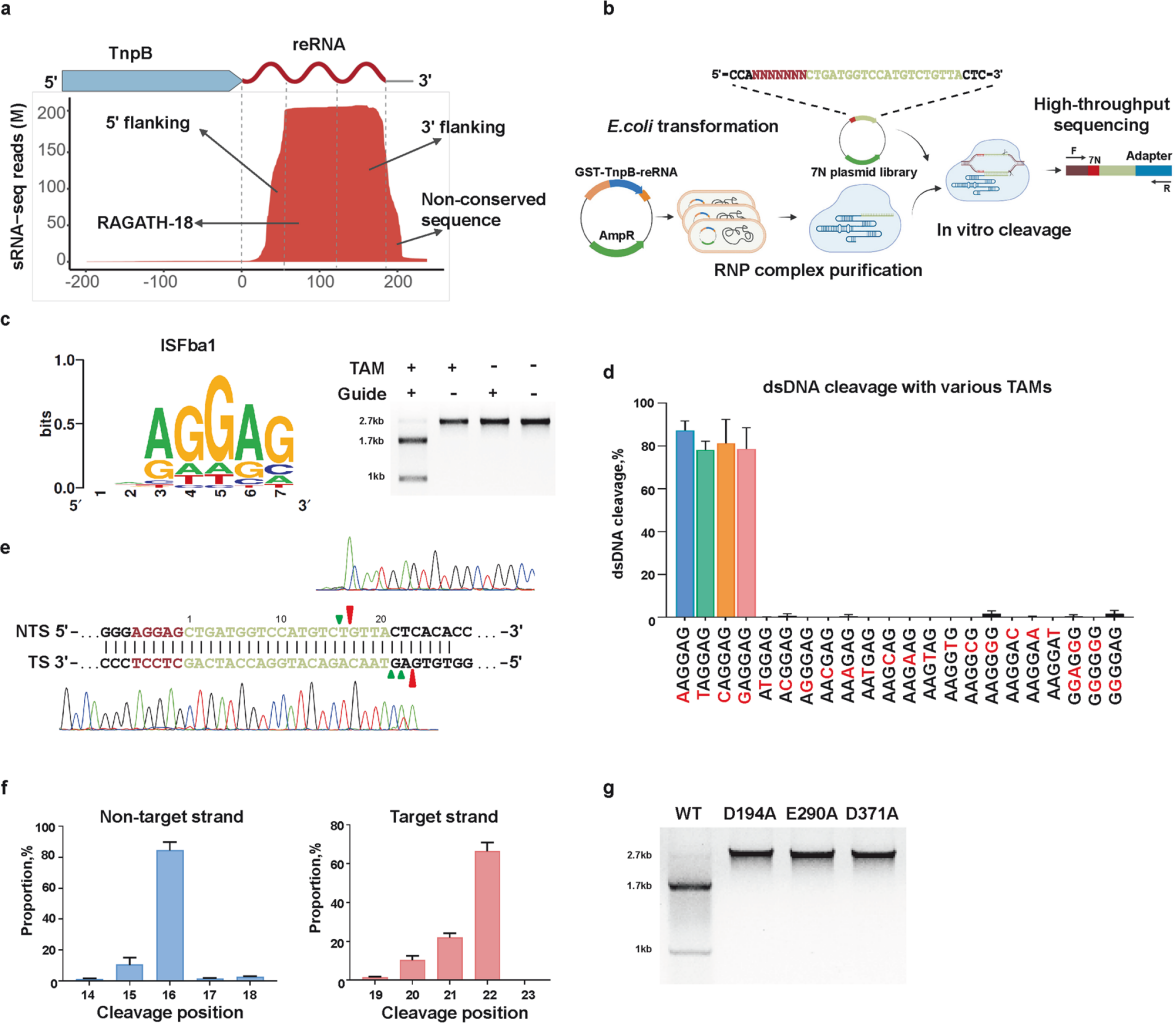
IS607 TnpBs are RNA-guided DNA endonucleases. **a** Mapping of small RNA sequencing data to the IS607 TnpB non-coding region in *Firmicutes bacterium*. **b** Scheme of the biochemical assay used to discover the TAM position and identity. **c** Weblogo of the identified ISFba1 TAM sequence (left panel); in vitro cleavage assay of ISFba1 TnpB using linearized dsDNA substrates (right panel). The agarose gel was visualized by ethidium bromide (EB) staining. TAM, Target adjacent motif; 2.7 kb, dsDNA substrate; 1.7 kb and 1 kb, cleavage product. Data shown are representative of three independent experiments. **d** Quantification of the in vitro cleavage assays mediated by ISFba1 TnpB using linearized dsDNA substrates with different TAM sequences. Data represent means ± SD of three biological replicates. **e** Run-off Sanger sequencing for the ISFba1 TnpB-cleaved plasmid. NTS, the non-targeted strand; TS, the target strand. The major and minor cleavage sites are indicated by red and green triangles. **f** The proportion of the non-target strand (left panel) and target strand (right panel) cleavage sites quantified by the high-throughput sequencing of the ISFba1 TnpB-cleaved results. Data represent means ± SD of three biological replicates. **g** In vitro cleavage assay of the wild-type ISFba1 TnpB (WT) and RuvC active site mutated ISFba1 TnpB (D194A, E290A, D371A) using linearized dsDNA substrates. The agarose gel was visualized by EB staining. Data shown are representative of three independent experiments.

The co-occurrence of RAGATH-18 RNAs and IS607 TnpBs suggests their functional co-operativity. We therefore investigated whether an IS607 TnpB protein and its adjacent RAGATH-18 RNA bind to each other by small RNA-sequencing (Supplementary information, Fig. S2a). A recombinant plasmid was constructed for co-expression of IS607 TnpB and its reRNA containing 5’ flanking, RAGATH-18, 3’ flanking and non-conserved sequences at its 3’ end from the *Firmicutes bacterium* AM43-11BH strain in *E. coli*. The protein purification results showed that the IS607 TnpB protein co-existed with a bound nucleic acid (Supplementary information, Fig. S2b). RNase treatment completely degraded the TnpB-bound nucleic acid. By contrast, the bound nucleic acid was insensitive to DNase treatment, indicating that the IS607 TnpB protein formed a complex with a RNA (Supplementary information, Fig. S2b). Small RNA sequencing showed that the TnpB-bound RNA contained 184 nucleotides, including a 37-nt 5’ flanking motif 13 nt downstream of gene encoding IS607 TnpB, a 73-nt RAGATH-18 motif, a 64-nt 3’ flanking motif, and a 10-nt non-conserved sequence adjacent to the 3’ flanking motif (Fig. 2a). These results demonstrate that the *Firmicutes bacterium* IS607 TnpB forms a specific RNA–protein complex with its adjacent reRNA.

Recent studies have shown that the TnpB protein of IS200/IS605 is a programmable endonuclease.^23,24^ We hypothesized that the IS607 TnpB protein may function similarly given its specific reRNA-binding activity. To test this hypothesis, we constructed a pUC19-based plasmid library containing seven randomized DNA nucleotides next to the upstream of a target sequence. For the programmable endonuclease of IS200/IS605 TnpB, the variable sequence at the 3’ end adjacent to the conserved flanking motif of the protein-bound RNA serves as a guide sequence for targeting DNA substrates.^23,24^ Inspired by the effective guide sequence length of existing programmable endonucleases, such as CRISPR/Cas9, we therefore replaced the variable sequence of the *Firmicutes bacterium* reRNA with a 20-nt sequence capable of targeting the plasmid library. The hepatitis delta virus (HDV) self-cleaving ribozyme was used to remove the sequence at the end of transcribed *Firmicutes bacterium* reRNA. Based on the sequence similarity clustering and taxonomy (up to 6 proteins per species), 43 TnpBs from 41 bacterial strains of 25 different species (Supplementary information, Table S2) were selected to test the dsDNA cleavage activity in vitro. We transformed the expression plasmids of TnpBs into *E. coli*, purified RNP complexes to cleave the constructed plasmid library in vitro, followed by high-throughput sequencing (Fig. 2b). The high-throughput sequencing results showed that the purified RNP complexes had reRNA-guided dsDNA cleavage activity, and revealed their target-adjacent motif (TAM)^23^ sequences (Supplementary information, Fig. S2c).

Sequencing data analysis showed that 23 IS607 TnpBs from 22 bacterial strains of 17 different species cleaved dsDNAs with a TAM sequence of 5’-AGGAG (18/22 strains) or 5’-GAGGG (4/22 strains) (Fig. 2c; Supplementary information, Fig. S2c). To verify an essential role of the TAM sequences in the endonuclease activity of the IS607 TnpB systems, we purified the IS607 TnpB protein from *Firmicutes bacterium* AM43-11BH (ISFba1 TnpB, 387 aa) and assayed its DNase activity with different dsDNA substrates. Consistent with the sequencing data (Supplementary information, Fig. S2c), ISFba1 was fully active in cleaving dsDNA with 5’-AGGAG as the TAM sequence (Fig. 2c). Single mutations of any of these five TAM nucleotides resulted in complete loss of the dsDNA cleaving activity (Fig. 2d), indicating that the TAM sequence for ISFba1 TnpB system is highly specific. Sanger sequencing revealed that the ISFba1 TnpB-cleaved products formed a 5’-overhang end at the TAM-distal region (Fig. 2e), and most of the cleavage sites were located at 22 nt (> 60%) of the target strand and 16 nt (> 80%) of the non-target strand (Fig. 2f). This activity was dependent on the predicted RuvC active site (D194, E290 and D371) of ISFba1 TnpB (Fig. 2g; Supplementary information, Fig. S2d), indicating that RuvC is responsible for cutting both strands of DNA substrates. This is reminiscent of Type V CRISPR-Cas12 family nucleases.^23,42^ BLASTP^43^ analysis showed that the similarity between IS607 TnpB family effectors and previously reported active TnpB^23,24^ is less than 35%, and the regions of similarity were primarily concentrated within the RuvC domain. Together, these results demonstrate that ISFba1 TnpB is a novel TAM-dependent DNA endonuclease and cleaves dsDNA substrates in an reRNA-dependent manner.

We biochemically characterized the compact ISFba1 TnpB by evaluating the effects of divalent metal ions, salt concentration, and temperature on the dsDNA cleavage activity of ISFba1 TnpB in vitro. Our results showed that the endonuclease activity is Mg^2+^-dependent; under the condition of Mn^2+^ or Ca^2+^, it possessed low DNase activity (Supplementary information, Fig. S3a, b), and it achieved the highest endonuclease activity with 25–100 mM NaCl at the optimum temperature of 37 °C (Supplementary information, Fig. S3c, d). Screen of different sizes of the guide sequence of the *Firmicutes bacterium* reRNA showed that 20 nt is an optimal length for the DNase activity of ISFba1 TnpB (Supplementary information, Fig. S3e).

The *trans*-cleavage activity toward non-target single-stranded DNA (ssDNA) has been observed in RuvC domain-containing Cas12 family nucleases and adapted for detecting nucleic acids.^44–46^ To investigate whether the ISFba1 TnpB system has the *trans*-cleavage activity, we performed a cleavage assay using 5’-FAM-labeled non-target ssDNA as the substrate and a target ssDNA as an activator. The results showed that ISFba1 TnpB had *trans*-cleavage activity toward the non-target ssDNA in the presence of the ssDNA activator, as observed for CRISPR-Cas12a^45,47^ (Supplementary information, Fig. S2e).

### DNA interference activity of IS607 TnpBs in bacteria

**Fig. 3:**
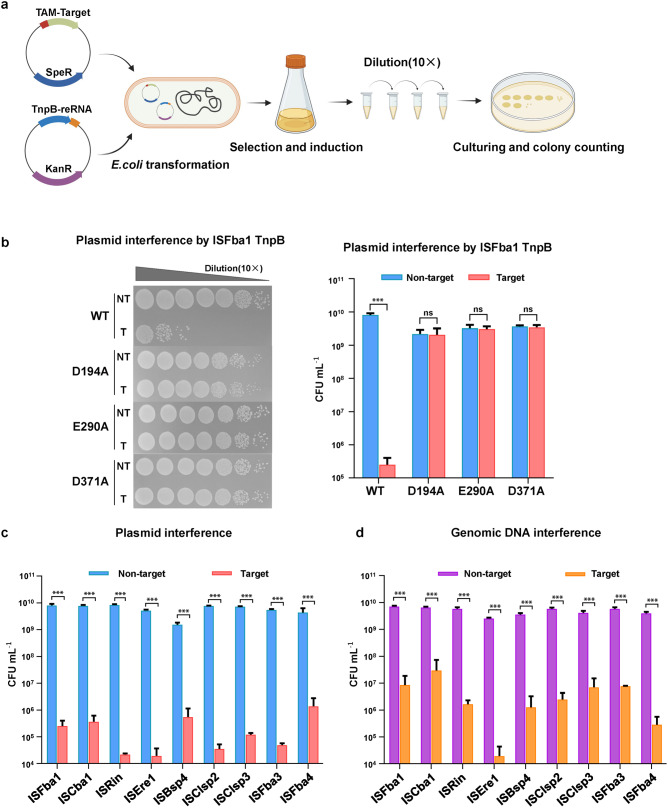
IS607 TnpB systems have the DNA interference capability in bacteria. **a** Scheme of plasmid interference in *E. coli* mediated by the IS607 TnpB systems. KanR, kanamycin resistant; SpeR, spectinomycin resistant. **b** Plasmid interference assay of wild-type ISFba1 TnpB (WT) and RuvC active site mutated ISFba1 TnpB (D194A, E290A, D371A) using 5’-AGGAG TAM. The culture samples were serially diluted (10×) and selected by the media supplemented with kanamycin (Kan) and spectinomycin (Spe) (left panel). NT, non-target control group; T, target group. Data shown are representatives of three independent experiments. Quantification of the corresponding plasmid interference assay (right panel). Data represent means ± SD of three biological replicates. Two-tailed unpaired *t*-test: ***P < 0.001, ns, not significant. **c** Quantification of the plasmid interference by TnpBs of ISFba1, ISCba1, ISRin, ISEre1, ISBsp4, ISClsp2, ISClsp3, ISFba3 and ISFba4 using 5’-AGGAG TAM. The corresponding serial dilution assay is shown in Supplementary information, Fig. S3f. The detailed information of the IS607 TnpB proteins is provided in Supplementary information, Table S2. Data represent means ± SD of three biological replicates. Two-tailed unpaired *t*-test: ***P < 0.001. **d** Quantification of the genomic DNA interference mediated by TnpBs of ISFba1, ISCba1, ISRin, ISEre1, ISBsp4, ISClsp2, ISClsp3, ISFba3 and ISFba4 in *E. coli*. The corresponding serial dilution assay is shown in Supplementary information, Fig. S3h. Data represent means ± SD of three biological replicates. Two-tailed unpaired *t*-test: ***P < 0.001.

The results above demonstrate that IS607 TnpBs are programmable endonucleases. We then investigated whether the IS607 TnpB systems have DNA interference activity in bacteria. To this end, we performed plasmid and endogenous genomic DNA interference assays in *E. coli*. For the plasmid interference experiment, plasmids expressing 23 IS607 TnpB RNP complexes from 22 bacterial strains of 17 different species were individually co-transformed with a spectinomycin-resistant plasmid containing 5’-AGGAG or 5’-GAGGG TAM and 20-nt target sequence into *E. coli*. The clones were selected on plates containing kanamycin and spectinomycin by a 10-fold gradient dilution after induction of expression (Fig. 3a). Compared with the non-target control, the co-transformation of plasmids encoding 9 IS607 TnpB members from 8 strains of 6 different species and plasmids with target sequences led to more than 10^4^-fold colony reduction, demonstrating robust plasmid interference ability of these IS607 TnpB systems in *E. coli* (Fig. 3b, c; Supplementary information, Fig. S3f, g and Table S2). Inactivation of the RuvC active site (D194, E290 and D371) prevented ISFba1 TnpB from generating DNA interference (Fig. 3b), indicating that the DNA interference activity of ISFba1 TnpB is dependent on its enzymatic activity. For the genomic DNA interference assays, IS607 TnpB RNP complex expressing plasmids with a 20-nt guide sequence targeting *E. coli* genome sites were constructed and transformed into *E. coli* and selected by kanamycin. A 10-fold dilution titration assay showed that 12 IS607 TnpB members from 11 strains of 7 different species effectively mediated the genomic DNA interference and inhibited growth of the bacterium *E. coli* (Fig. 3d; Supplementary information, Fig. S3h, i and Table S2). These data collectively demonstrate that the IS607 TnpB systems have the activity of cleaving dsDNA substrates in bacteria.

### IS607 TnpB-mediated genome editing in human cells

**Fig. 4:**
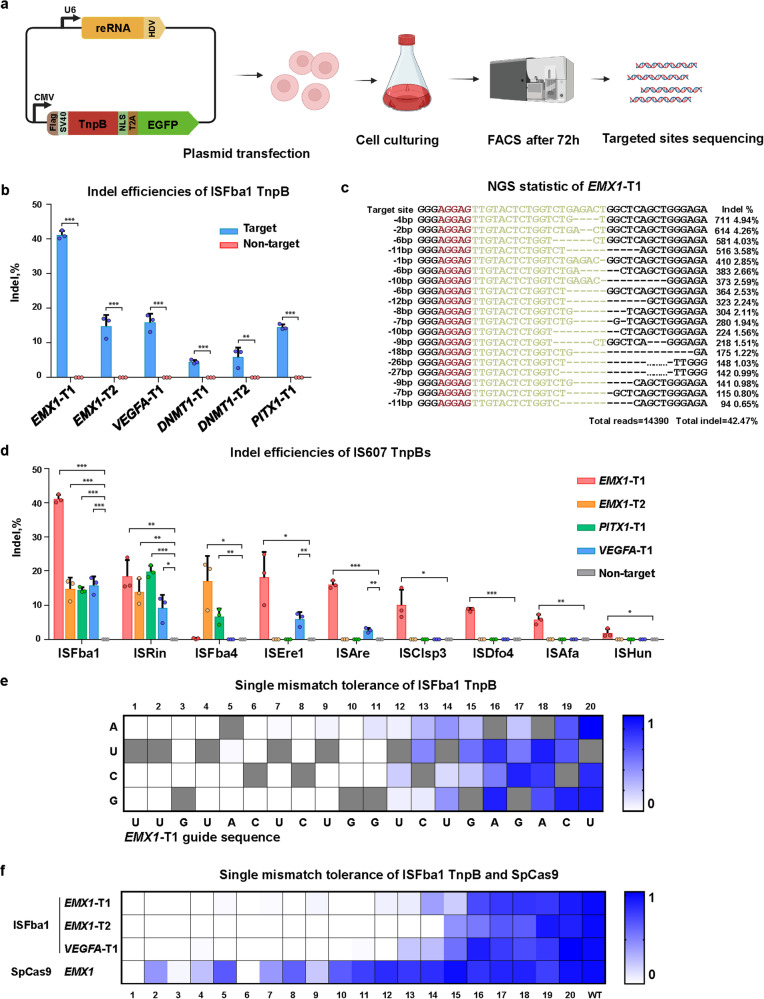
IS607 TnpB-mediated genome editing in human cells. **a** Scheme of the human cell line (HEK293F) genome-editing experiment. **b** The indel efficiencies of ISFba1 TnpB on endogenous loci *EMX1*-T1, *EMX1*-T2, *VEGFA*-T1, *DNMT1*-T1, *DNMT1*-T2 and *PITX1*-T1 in HEK293F cells, determined by next-generation sequencing (NGS). Data represent means ± SD of three biological replicates. Two-tailed unpaired *t*-test: ***P < 0.001, **P < 0.01. **c** NGS statistic of indel efficiency of ISFba1 TnpB on the endogenous locus *EMX1*-T1. **d** The indel efficiencies mediated by TnpBs of ISFba1, ISRin, ISFba4, ISEre1, ISAre, ISClsp3, ISDfo4, ISAfa and ISHun on the endogenous loci. Data represent means ± SD of three biological replicates. Two-tailed unpaired *t*-test: ***P < 0.001, **P < 0.01, *P < 0.05. The detailed information of different IS607 TnpB proteins is provided in Supplementary information, Table S2. The guide sequences are provided in Supplementary information, Table S5. **e** Heatmap representation of the ISFba1 TnpB cleavage efficiency with 60 single-nucleotide-mutated guide RNAs for *EMX1*-T1 target site. The identities of single base pair substitutions are indicated on the left; original guide sequence is shown at the bottom and highlighted in the heatmap (gray squares). The cleavage efficiencies were monitored by high-throughput sequencing. Modification efficiencies (increasing from white to blue) are normalized to the original guide sequence. 1–20, mismatch position 1–20. **f** Heatmap of the single mismatch tolerance of ISFba1 TnpB on the *EMX1*-T1, *EMX1*-T2, *VEGFA*-T1 target sites and SpCas9 on the *EMX1* target site. 1–20, mismatch position 1–20; WT, original guide sequence. Data shown are representative of three independent experiments. The guide sequences are provided in Supplementary information, Table S5.

Having established that the IS607 TnpB systems cleave both plasmid and genomic DNA in bacteria, we further explored their genome editing ability in mammalian cells. We therefore constructed plasmids encoding 37 EGFP-linked IS607 TnpB proteins and their corresponding reRNAs carrying 20-nt genomic DNA-targeting sequences. These 37 TnpBs included 14 TnpBs with low homology (< 50% sequence similarity) to ISFba1 TnpB within the same species or with high homology (> 80% sequence similarity) to ISFba1 TnpB in different species and the 23 TnpBs that had been tested in bacteria (Supplementary information, Table S2). The processed TnpB contained a SV40 nuclear localization peptide at the N-terminus and a nuclear localization signal peptide at the C-terminus. The recombinant plasmids were transfected into 293F cells. After 72-h transfection, 293F cells expressing green fluorescent protein were sorted out and the genomic DNA of these cells was extracted for high-throughput sequencing of the targeted sites. The DNA editing efficiencies of the IS607 TnpBs expressed in the human cells were quantified by the frequencies of insertions or deletions (indels) generated on the target sites (Fig. 4a). The data from the assays showed that ISFba1 TnpB displayed editing efficiency at the *EMX1* and *VEGFA* loci: 42.5% at *EMX1-*T1, and 17.0% at *EMX1-*T2 and 17.9% at *VEGFA*-T1 (Fig. 4b, c). These results indicate that ISFba1 TnpB has comparable genome editing activity with SpCas9 and LbCas12a when they were originally developed.^15,16^ IS607 TnpBs from other strains also demonstrated DNA editing activity, albeit with lower efficiency (Fig. 4d).

We next investigated substrate specificity of ISFba1 TnpB using the *EMX1* and *VEGFA* sites in 293F. A set of 60 different guide RNAs were generated containing all possible single-nucleotide substitutions of nucleotides in positions 1–20 adjacent to the 5’-AGGAG TAM (Fig. 4e). Single mismatch between the guide and 1–13 bp of target sequence resulted in loss or significant impairment of the endonuclease activity of ISFba1 TnpB (Fig. 4f). In contrast to ISFba1 TnpB, SpCas9 tolerated more single mismatches between the guide and target sequences (Fig. 4f).^48,49 ^Consistently, ISFba1 TnpB shows more sensitivity to double mismatches when compared with SpCas9 (Supplementary information, Fig. S3j). Based on the *EMX1-*T1 site described above, we computationally selected 9 candidate off-target sites in the human genome with a 5’-AGGAG TAM. High-throughput sequencing results revealed that there was no gene editing at all predicted off-target sites (Supplementary information, Fig. S3k). These results indicated that the IS607 TnpB system can be harnessed as a programmable genome editing tool with high specificity.

### Cryo-EM structure of ISFba1 TnpB–reRNA–target DNA

**Fig. 5:**
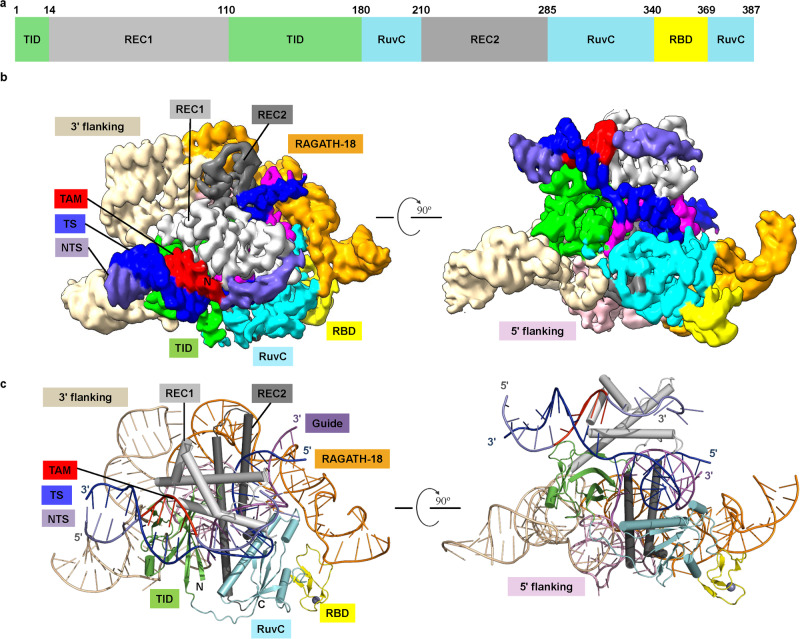
Cryo-EM structure of the ISFba1 TnpB–reRNA–dsDNA complex. **a** Domain organization of ISFba1 TnpB. TID, TAM-interacting domain; REC, recognition domain. TID and RuvC domains are separated into two and three segments, respectively. **b**, **c** Cryo-EM reconstruction at 3.0 Å (**b**) and cartoon representations of the ISFba1 TnpB–reRNA–dsDNA complex (**c**).

To elucidate the structural mechanism of reRNA-guided DNA targeting by the miniature IS607 TnpB systems, we focused on the ISFba1 TnpB system, which has high DNA cleavage activity both in vitro and in vivo. We determined the structure of a ternary complex comprising ISFba1 TnpB (D371A catalytic mutant), a 207-nt reRNA containing a 20-nt guide segment, a 36-nt target DNA strand and a 20-nt non-target DNA strand with a 5’-AGGAG TAM using cryo-EM at resolution of 3.0 Å (Fig. 5a–c; Supplementary information, Figs. S4, S5 and Table S3). The resultant cryo-EM density map allowed us to build the atomic model of the whole ternary complex (Fig. 5b), except for three residues at the N-terminus of the ISFba1 TnpB protein, and flexible loop regions in the reRNA and the target DNA. The structure revealed that ISFba1 TnpB and reRNA form a complex with 1:1 stoichiometry (Fig. 5a–c). ISFba1 TnpB consists of an amino-terminal TAM-interacting domain (TID) and a non-target-strand recognition (REC1) domain, a target-strand recognition (REC2) domain, a reRNA-binding domain (RBD), and a RuvC domain at its carboxy-terminus. The guide RNA–target DNA heteroduplex binds to the central channel formed by TID and REC1 from one side, and the REC2 and RuvC domains from the other side (Fig. 5a–c). The architecture organization mode of the ISFba1 TnpB ternary complex is distinct from those of Cas9, Cas12a and Cas12f^50–52^ (Supplementary information, Fig. S6). One significant difference is that ISFba1 TnpB possesses much smaller REC domains. Additionally, the reRNA in the ISFba1 TnpB system adopts a strikingly different fold from the RNAs in the other three systems. In ISFba1 TnpB system, the reRNA plays a scaffolding role in organizing the complex via stabilizing the conformation of the ISFba1 TnpB effector protein for targeting the dsDNA substrate. This results in solvent exposure of a large portion of the bound reRNA. By contrast, a much lower percentage of the bound RNAs in Cas9 and Cas12a is solvent exposed.^50,53,54^

### ReRNA architecture

**Fig. 6:**
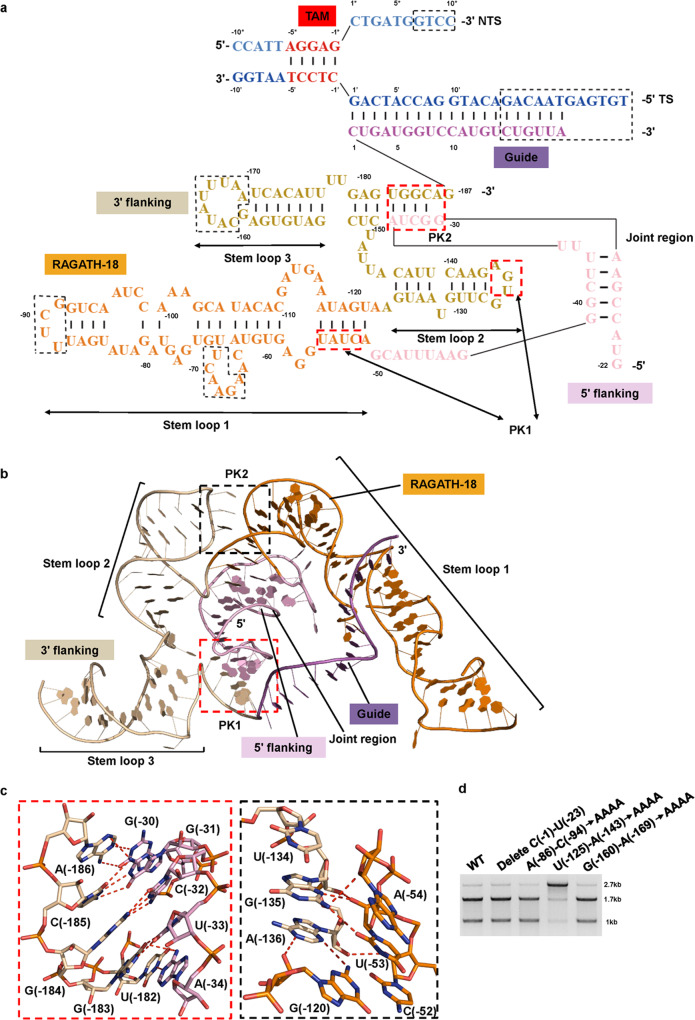
Structural organization of the reRNA. **a** Schematic representation of the reRNA and target DNA. The disordered regions are enclosed in black dashed boxes. The pseudoknot regions are enclosed in red dashed boxes. The nucleotide colors are the same as the cartoon representations in Fig. 5c. TS, target strand; NTS, non-target strand; TAM, target adjacent motif; PK, pseudoknot. **b** Structure of the reRNA scaffold. **c** The conformation of PK1 (right panel) and PK2 (left panel) **d** In vitro cleavage of dsDNA substrates by ISFba1 TnpB RNP with full-length reRNA or truncated or mutant reRNA. AAAA, four consecutive ATP linker used to replace the nucleotides in the mutant regions. The agarose gel was visualized by EB staining. Data shown are representative of three independent experiments.

The reRNA from *Firmicutes bacterium* consists of a guide sequence of 20 nt, which forms an RNA–DNA heteroduplex with the target DNA strand, and the 187-nt RNA scaffold. The RNA scaffold contains a 5’ flanking (G(–22)–G(–50)) motif, RAGATH-18 (A(–51)–A(–122)) motif and a 3’ flanking (A(–123)–G(–187)) motif. The latter can be divided into a joint region (G(–22)–G(–50)), three stem loops (SL1–3) and two pseudoknots (PK1 and PK2) (Fig. 6a–c). The R-loop region contains the 10-bp 5’-AGGAG TAM-containing dsDNA, the 14-bp RNA–DNA heteroduplex and 6-nt non-target DNA strand (Fig. 6a). The upper region of the 5’ flanking motif (C(–1)–U(–21)) and the loop regions of SL1 and SL3 are disordered in the cryo-EM structure, suggesting flexibility of these regions in solution. Deletion and mutations of these disordered regions had no detectable impact on IS607 TnpB-mediated DNA cleavage (Fig. 6d), suggesting that they are dispensable for the formation of the IS607 TnpB complex. The 5’ flanking motif is located at the central portion of the reRNA, adopting a curved conformation to interact with both the RAGATH-18 and the 3’ flanking motif (Fig. 6b). Notably, the looped-out bases G(–30)–A(–34) of the apical loop of the 5’ flanking motif base pair with U(–182)–A(–186) of the 3’ flanking motif, forming PK2 to further stabilize the reRNA structure (Fig. 6c, left panel). Mutations of the bases G(–30)–A(–34) impaired ISFba1 TnpB RNP complex formation (data not shown), highlighting the importance of PK2 for ISFba1 TnpB function. The RAGATH-18 motif adopts a supercoiled structure with a long stem loop emanating from the three-motif junction region. The 3’ flanking motif contains two stem loops, with U(–134)–A(–136) flipping out from SL2 to form PK1 with bases of A(–54)–U(–53) and G(–120) of the RAGATH-18 RNA, further stabilizing the reRNA scaffold structure (Fig. 6c, right panel). Mutations disrupting base pairs in PK1 abolished the DNA cleavage activity of ISFba1 TnpB, supporting the functional importance of PK1 in the ISFba1 TnpB system (Fig. 6d).

### Recognition of the reRNA and target dsDNA by ISFba1 TnpB

**Fig. 7:**
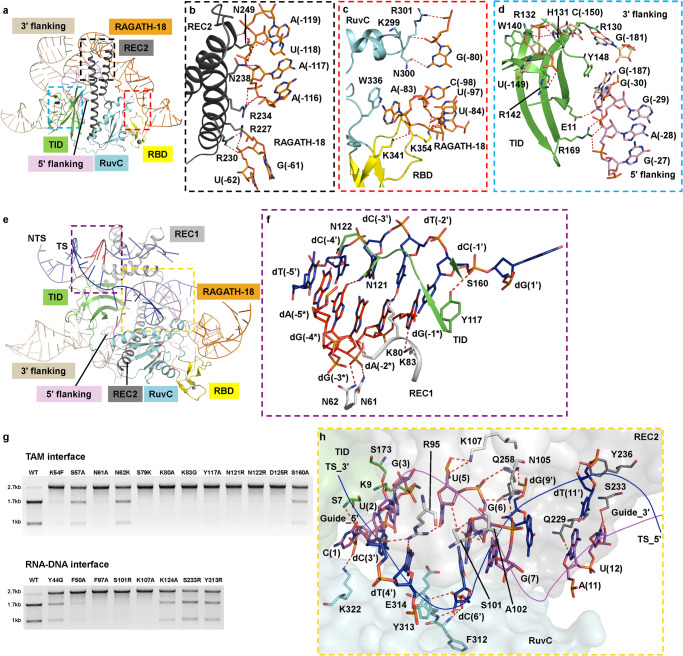
Recognition of the reRNA and target DNA. **a** Inset shows the location of zoomed structure in **b**–**d**. **b**–**d** reRNA recognition by REC2 (**b**), RuvC and RBD (**c**) and TID (**d**). **e** Inset shows the location of zoomed structure in **f**–**h**. **f** TAM recognition by the TID and REC1 domains. **g** In vitro cleavage of dsDNA substrates by ISFba1 TnpB RNP with wild-type (WT) or mutant ISFba1 TnpB protein. The agarose gel was visualized by EB staining. Data shown are representative of three independent experiments. **h** RNA–DNA heteroduplex recognition by the TID, RuvC and REC2 domains.

The REC2 (residues R227, R230, R234, N238 and N249), RuvC (residues K299, N300 and R301) and RBD (residues K341 and K354) domains pack against the sugar-phosphate backbone of the reRNA at one side (Fig. 7a–c; Supplementary information, Fig. S7), whereas TID is anchored on SL3 of the 3’ flanking and 5’ flanking motifs of the reRNA through polar interactions via R130, R132 and R142 (Fig. 7d; Supplementary information, Fig. S7). In addition, H131 and Y148 of TID and W336 of RuvC stack against the nucleobases C(–150), G(–187) and A(–83) of reRNA, respectively, contributing to the interaction between ISFba1 TnpB and the reRNA scaffold. Sequence analysis of IS607 TnpBs from different bacterial species showed that these reRNA-recognizing residues of ISFba1 TnpB are conserved (Supplementary information, Fig. S8). These results suggest conserved reRNA-binding and consequently programmable endonuclease activities of the IS607 TnpB effector proteins. This conclusion is supported by our observation that many IS607 TnpB proteins displayed reRNA-guided dsDNA cleavage activity (Supplementary information, Fig. S2c).

The much smaller sizes of IS607 TnpB effector proteins compared to other dsDNA-targeting CRISPR-Cas effector proteins suggest a unique mechanism of dsDNA substrate recognition and R-loop structure formation. The 5’-AGGAG TAM-containing duplex is specifically recognized by REC1 and TID (Fig. 7e, f). The nucleobases of dG(–1*), dG(–4*) and dA(–5*) of TAM form hydrogen-bonding interactions with K83, N121 and N122 of TID (Fig. 7f), respectively. Furthermore, the nucleobases of dT(–2’) and dC(–3’) base pair with dA(–2*) and dG(–3*) of TAM, and form hydrogen bonds with K80 and N121, respectively. The backbone phosphate of dA(–2*) is also recognized by N61 and N62 in REC1 (Fig. 7f), further stabilizing the TAM segment. Mutations of the TAM-interacting residues strongly reduced the dsDNA cleavage activity of ISFba1 TnpB (Fig. 7g), confirming the significance of TAM in recognition of dsDNA. The backbone phosphate group of dC(–1’) in the target strand hydrogen bonds with the side chains of Y117 and S160 in TID (Supplementary information, Fig. S7), facilitating formation of the guide RNA–target DNA heteroduplex. The Y117A and S160A mutations reduced the endonuclease activity of ISFba1 TnpB, albeit with different efficiency (Fig. 7g), supporting a critical role of these two residues in the formation of the RNA–DNA heteroduplex. Taken together, these results explain the specific recognition of the 5’-AGGAG TAM by ISFba1 TnpB and provide the structural base for expanding the TAM preference through altering ISFba1 TnpB and TAM interactions.^55–57^

The guide RNA–target DNA heteroduplex is bound by the REC1 domain from the top, and the RuvC and REC2 domains from the bottom through polar interactions with its sugar-phosphate backbone. By comparison, the non-target strand is primarily bound by the REC1 domain (Fig. 7f; Supplementary information, Fig. S7). At the TAM-distal end, the nucleotides dG(15’)–dT(20’) of the target strand and the nucleotides G(7*)–C(10*) of the non-target strand are poorly defined. By comparison, base pairs 15–20 from the TAM-distal end are well defined but make no contact with the IS607 TnpB protein. The TAM-proximal end (base pairs 1–7) of the heteroduplex is recognized by the REC1 and RuvC domains, whereas the middle portion (base pairs 8–14) of the heteroduplex is bound by the REC2 domain (Fig. 7f, h). This is distinct from the Cas9 and Cas12a systems^42,50,51^ in which the entire RNA–DNA heteroduplex from the TAM-proximal to TAM-distal ends is recognized by both the REC and RuvC domains (Supplementary information, Fig. S6), resulting in more extensive interactions between the heteroduplex and Cas9 or Cas12a. This may explain higher tolerance of mismatches^48,58^ compared to the ISFba1 TnpB system (Fig. 4e, f). In this regard, our structural observation revealed that, as compared with Cas9, the reduced contacts between RNA–DNA heteroduplex and the REC domain of ISFba1 TnpB support that ISFba1 TnpB may target a dsDNA substrate stringently despite its miniature size.

Specific interactions between the non-target strand and ISFba1 TnpB are also established in the RNP complex. The nucleobases of dA(4*) and dG(6*) in the non-target strand form stacking interactions with conserved F50/F97/Y44 and F103 (Supplementary information, Fig. S7), respectively, further reinforcing recognition of the RNA–DNA heteroduplex by ISFba1 TnpB. In addition to the stacking interactions, the nucleobase of dC(1*) forms two hydrogen bonds with the hydroxyl group of S52. Consistent with the structural observation, mutations of these residues involved in the recognition of the non-target strand and heteroduplex resulted in severe or modest reductions in the dsDNA cleavage activity of ISFba1 TnpB (Fig. 7g), indicating a critical role for ISFba1 TnpB recognition of non-target strand in dsDNA cleavage.

Recently, the structure of ISDra2 TnpB from IS200/IS605 was reported by Nakagawa et al.^59^ and Sasnauskas et al.^60^ Our structural comparison reveals that both ISFba1 and ISDra2 TnpB adopt a bi-lobed (Rec and Nuc) architecture twisting around the RNA–DNA heteroduplex (Supplementary information, Figs. S6a, S9). The obvious structural differences between ISFba1 and ISDra2 occur in the REC and RBD domains of ISFba1. In addition, ISDra2 does not contain the SL3 binding helix of TID found in ISFba1, consequently, its corresponding reRNA lacks the SL3 motif observed in reRNA of ISFba1 system. Furthermore, both the SL1 of reRNA and its interacting domain RBD of ISFba1 system are absent in the ISDra2 system (Supplementary information, Figs. S6b, S9), indicating the different structural arrangements. Interestingly, the TAM recognition model of ISFba1 TnpB differs significantly with ISDra2 TnpB. The residues K83 and N121 of ISFba1 TnpB form hydrogen bonds with the nucleobases of dG(–1*) and dG(–4*), respectively (Fig. 7f). While, the corresponding residues Q80 and T123 of ISDra2 TnpB directly interacts with the nucleobases of dT(–1*) and dT(–4*), respectively. The structural information indicates that ISDra2 TnpB more favors AT-rich TAM, while ISFba1 TnpB favors G-rich TAM.

## Discussion

In the current study, we provide biochemical evidence that the ISFba1 TnpB forms a complex with its reRNA and acts as an reRNA-guided DNase in vitro. More importantly, ISFba1 TnpB displayed DNA interference activity in both *E. coli* and human cells. Cryo-EM analysis of an ISFba1–reRNA–dsDNA substrate complex revealed that amino acids involved in ISFba1 recognition of the reRNA and dsDNA are highly conserved among different IS607 TnpB effectors co-existing with RAGATH-18 RNA (Supplementary information, Figs. S7, S8). This suggests that reRNA-guided dsDNA cleavage activity is conserved among those IS607 TnpBs, a conclusion that is confirmed by our biochemical and in vivo data (Figs. 2–4; Supplementary information, Figs. S2, S3). Collectively, our results report that IS607 TnpBs, which co-exist with RAGATH-18 RNA, are a large family of programmable DNases found in many different bacterial species. To the best of our knowledge, these IS607 TnpBs with an average size of 390 aa (330–433 aa) represent the smallest programmable endonucleases with activity in human cells identified thus far. Our study thus opens new opportunities of employing programmable endonucleases for gene editing.

Compared to the widely used genome editing effectors Cas9 and Cas12a with sizes of about 1200–1400 aa, IS607 TnpB effectors have much smaller sizes of about 330–433 aa. This is presumably conducive to overcoming the delivery challenges that gene editing technology face.^61^ Interestingly, a guide sequence of ∼20 nt appears to be optimal for the dsDNA cleavage activity of IS607 TnpB effectors (Supplementary information, Fig. S3e) despite that they have much smaller sizes as compared with Cas9 and Cas12a. However, ISFba1 TnpB appears to form fewer contacts with the RNA–DNA heteroduplex than SpCas9.^50^ High-throughput sequencing evidence showed that the ISFba1 TnpB system is more sensitive to base mismatches of the guide RNA–DNA heteroduplex (Fig. 4e, f; Supplementary information, Fig. S3j, k) than the SpCas9 system.^49^ This further supports the idea that tolerance of RNA–DNA mismatches inversely correlates with the interactions between Cas9 and RNA–DNA heteroduplex as shown by the data from our and other groups.^48,62^ Recently, the compact CRISPR-AsCas12f1 (422 aa, with ~200-nt sgRNA) was discovered as a potential genome editing tool.^63^ The structural comparison (Supplementary information, Fig. S6) confirms the similarity between AsCas12f and IS607 TnpB. In contrast with ISFba1, Cas12f RNP forms a homodimer and binds to a single copy of crRNA/*trans*-activation crRNA hetrodimer.^52^ In addition, the IS607 TnpB and Cas12f nucleases demonstrate distinct TAM and PAM sequence requirement for dsDNA cleavage.

Recent studies identified transposon-associated RNA-guided endonucleases TnpB and IscB of IS200/IS605.^23,24^ Like Cas12f,^64^ ISDra2 TnpB (408 aa)^23^ and KraIscB-1 (432 aa)^24^ have smaller sizes as compared with Cas9 and Cas12a. However, there are important differences between TnpB of IS607 family co-existing with RAGATH-18 RNA and IS200/IS605 families. First, the guide RNAs of IS200/IS605 IscB and TnpB are overlapped with IscB and TnpB protein coding sequences, respectively, while the reRNA of the IS607 TnpB is totally independent of the protein coding sequence, which is convenient for the separation of the two elements for genome editing applications. Second, IS607 TnpB co-existing with RAGATH-18 RNA and IS200/IS605 IscB and TnpB have different TAM sequence requirements. Third, the structural comparison between ISFba1 and ISDra2 TnpB (8EXA)^60^ complexes revealed that significant structural differences exist in all three stem loop motifs of reRNA, and in TID, REC, and RBD domains of ISFba1 TnpB protein (Supplementary information, Figs. S6a, S9), respectively. Taken together, the structural comparison in combination with the bioinformatic data strongly support that IS607 TnpBs and IS200/IS605 TnpBs are closely related but belong to different clades.

Different engineering strategies including protein and guide RNA engineering have been successfully used to optimize CRISPR-Cas systems.^65,66^ Based on the small size and high sensitivity to base mismatches between the guide and target DNA, the IS607 TnpB systems offer a huge advantage for engineering to improve their precision, specificity, and versatility. TAM optimization through IS607 TnpB protein engineering to expand the scope of use is worth exploration in the future. Many members of the systems are highly conserved in different bacterial species. However, our in vivo data showed that their DNA interference activities vary significantly in *E. coli* (Fig. 3c, d) and human cells (Fig. 4d, e). Currently, the mechanisms underlying the differences remain unknown. It could be that differences in IS607 TnpB effector proteins or their cognate reRNAs or both are important. Additionally, half-lives of the IS607 TnpB systems could also impact their gene editing efficiency, in particular because the solvent-exposed portions of reRNAs could render TnpB RNPs susceptible to degradation in cells. Although the detailed mechanisms remain to be investigated, the IS607 TnpB systems provide diverse new templates for engineering of compact enzymes for gene editing.

Three members of the RAGATH RNA family, including the Hammerhead variants, the HDV variants, and the Twister sister, have been shown to be ribozymes,^67^ but the biological and biochemical functions of the remaining 33 family members remain unknown. Among them, RAGATH-18 RNA is the most abundant member found in different microorganisms.^28^ Our data revealed a scaffolding role for RAGATH-18 RNAs in binding the IS607 TnpB nucleases and guiding them to target dsDNA substrates for cleavage. Similarly, the IS200/605 TnpB nucleases, members of the IS transposon-encoded nuclease family, have been shown to exhibit RNA-induced nuclease activity.^24^ Based on these findings, it will be of interest to investigate whether other members of the IS transposase family may have a similar activity. We identified conserved genomic regions that potentially represent the LE and RE of 23 IS607 TnpB systems with strong dsDNA interference activity by extending both the upstream and downstream sequences of these systems by 2000 bp. Subsequently, AG-rich cognate TAMs were identified adjacent to the putative LE, matching with the experimentally identified TAM that can be recognized by IS607 TnpB systems. These results are consistent with the models for IS605 TnpB function proposed by the Siksnys lab^23^ and experimentally confirmed by the Sternberg lab,^68^ indicating the putative function of IS607 TnpB in the life cycle of the mobile element.

## Methods

### Plasmid construction

Genes encoding IS607 TnpB proteins and the non-coding RNA regions of the IS607 TnpB loci were synthesized from Genewiz. To purify the IS607 TnpB RNP complex from bacteria, a pGEX-6P-1-based plasmid was constructed to carry a full-length IS607 TnpB protein and downstream non-coding RNA region with a 20-nt guide sequence. Mutations of IS607 TnpB systems were created by circular polymerase extension cloning (CPEC).^69^ A plasmid library that was constructed as previously described^23,70^ and contained 7-bp randomized nucleotides located at the immediate 5’ upstream of the target sequence was used to characterize TAM. For in vitro dsDNA cleavage assay, pUC19-based plasmids carrying different TAMs and 20-bp target sequences were constructed via CPEC. For in vivo DNA interference in *E. coli*, a pET28a-based plasmid was constructed to encode the IS607 TnpB protein and downstream non-coding region with a 20-nt guide sequence. The pcs101-ori-based plasmids were constructed with different TAMs and 20-bp target sequences. For human genome editing, a plasmid encoding CMV-driven IS607 TnpB protein (human codon-optimized, N-terminal Flag and SV40 tagged, C-terminal NLS tagged) fused with EGFP was constructed with a U6-driven reRNA cassette. Different spacer insertions were constructed via CPEC. The sequences of the IS607 TnpB protein and reRNA are listed in Supplementary information, Table S2 and detailed sequence information for the plasmids used in this study is provided in Supplementary information, Table S4.

### Expression and purification of the IS607 TnpB RNP complexes

The IS607 TnpB RNP complexes were expressed in *E. coli* C43 (DE3) cells induced by 0.3 mM isopropyl β-D-1-thiogalactopyranoside (IPTG) at 16 °C. After overnight induction, the cells were collected by centrifugation, resuspended with resuspension buffer (25 mM Tris-HCl, pH 8.0, 1 M NaCl, 3 mM DTT) supplemented with 2 mM protease inhibitor phenylmethanesulphonylfluoride (PMSF, Sigma). The cells were lysed by sonication and cell debris was removed by centrifugation at 23,000× *g* for 40 min at 4 °C. The supernatant lysate was first flowed through glutathione sepharose 4B (GS4B) beads (GE Healthcare). The beads were then washed with buffer A (25 mM Tris-HCl, pH 8.0, 1.5 M NaCl, 3 mM DTT) and the bound IS607 TnpB RNP complexes were cleaved by precision protease in buffer B (25 mM Tris-HCl, pH 8.0, 300 mM NaCl, 3 mM DTT) overnight at 4 °C to remove the GST tag. The eluted IS607 TnpB RNP complexes were concentrated to 2 mL and further fractionated by size-exclusion chromatography (Superdex 200 100/300 GL, GE Healthcare) with buffer C (25 mM Tris-HCl, pH 8.0, 150 mM NaCl, 3 mM DTT, 5 mM MgCl_2_) via FPLC (AKTA Pure). Peak fractions were merged and concentrated to 5–10 mg/mL. The IS607 TnpB RNP complexes were used for subsequent small RNA sequencing, TAM characterization and DNA cleavage assay. To obtain the ISFba1 TnpB–reRNA–target DNA complex for cryo-EM analysis, a 36-nt target DNA strand and a 20-nt non-target DNA strand with a 5’-AGGAG TAM were mixed in a 1:1.25 molar ratio (final concentration of 80 μM) in annealing buffer (100 mM NaCl, 25 mM Tris-HCl, pH 8.0), hybridized by heating to 95 °C for 3 min, followed by slow cooling to room temperature. The ISFba1 TnpB–reRNA complex was incubated with pre-hybridized dsDNA at the molar ratio of 1:1.5 at room temperature for 5 min and 4 °C for 30 min supplemented with 5 mM MgCl_2_. The complex was further fractionated by size-exclusion chromatography (Superdex 200 100/300 GL, GE Healthcare) with buffer C (25 mM Tris-HCl, pH 8.0, 150 mM NaCl, 3 mM DTT, 5 mM MgCl_2_) to remove excess dsDNA. Purity of the complex protein was monitored at all stages of the purification process using SDS-PAGE. The reRNA and DNA were monitored using 10% TBE-urea-PAGE gel electrophoresis and visualized by EB staining. Purified complexes were concentrated to 10–15 mg/mL for cryo-EM analysis. The oligo sequences used in this study are provided in Supplementary information, Table S5.

### The nuclease cleavage assay and small RNA sequence

For the DNase and RNase cleavage assay, 5 μg of the purified nucleic acid and protein complex was incubated with 2 U of DNase I (Sigma-Aldrich) and RNase I (Sigma-Aldrich) in buffer (25 mM Tris-HCl, pH 8.0, 150 mM NaCl, 3 mM DTT) at 37 °C for 30 min, respectively. The cleavage products were monitored with 10% TBE-urea-PAGE gel electrophoresis and visualized by EB staining.

The IS607 TnpB RNP complex was treated with 2 U of DNase I (Sigma-Aldrich) at 37 °C for 30 min and reRNA was subsequently purified by phenol/chloroform extraction. In brief, small RNA libraries were constructed using the NEB Next Multiplex Small RNA Library Prep Set for Illumina according to the manufacturer’s protocol. The library was sequenced on NovaSeq 6000 platform (Illumina) by Shanghai Personal Biotechnology Co. Ltd. (Shanghai, China).

### IS607 TnpB-mediated dsDNA cleavage detection and TAM characterization

IS607 TnpB RNP complexes were used to cleave the 7-bp randomized TAM library. Briefly, reactions were performed in a 50 μL system containing 50 nM IS607 TnpB RNP complex and 2 μg TAM library plasmid. Cleavage reactions were conducted at 37 °C for 60 min in cleavage buffer (25 mM Tris-HCl, pH 8.0, 50 mM NaCl, 3 mM DTT, 10 mM MgCl_2_). Reactions were stopped by incubating with 1 μL proteinase K (CWBiotech) at 56 °C for 5 min. Products were purified using Gel Extraction Kit (CWBiotech) following the manufacturer’s protocol. Cleaved DNA ends were repaired by adding 1 μL T4 DNA polymerase (Thermo Fisher Scientific), 1 μL 10 mM dNTP mix (Thermo Fisher Scientific) and incubating at 11 °C for 20 min. The reactions were then inactivated by heating them up to 75 °C for 10 min and 3’dA overhangs were added by incubating the reaction mixture with 1 μL A-tailing polymerase (TAKARA) and 1 μL 10 mM dATP (TAKARA) at 72 °C for 30 min. The A-tailing products (100 ng) were ligated with a dsDNA adapter containing a 3’-dT overhang (100 ng) at 16 °C for 1 h using 1 μL T4 DNA ligase (TAKARA) in 20 μL reaction volume. After ligation, the adapter-bearing cleavage products were PCR amplified to obtain sequences required for deep sequencing and subjected to Illumina sequencing offered by HI-TOM platform (http://121.40.237.174/Hi-TOM/) at State Key Laboratory of Rice Biology (China National Rice Research Institute, Chinese Academy of Agricultural Sciences, Hangzhou).^70,71^ The adapter sequences and primers used in this study are provided in Supplementary information, Table S5.

### In vitro DNA cleavage assay

For the dsDNA cleavage assay, the 2.7 kb linearized dsDNA substrate was obtained from pUC19-based target plasmids described above by PCR amplified. The primers are provided in Supplementary information, Table S5. Reactions were performed in a 20 μL system containing 100 nM IS607 TnpB RNP complex and 400 ng dsDNA at 37 °C for 30 min in cleavage buffer (25 mM Tris-HCl, pH 8.0, 50 mM NaCl, 3 mM DTT, 10 mM MgCl_2_). Reactions were stopped by incubating with 1 μL proteinase K (CWBiotech) at 56 °C for 5 min. Cleavage products were analyzed by agarose gel, visualized by EB staining and quantified using Image Quant software (GE Healthcare).

For run-off Sanger Sequencing, 1 μg pUC19-based target plasmid was incubated with 100 nM IS607 TnpB RNP complex in cleavage buffer (25 mM Tris-HCl, pH 8.0, 50 mM NaCl, 3 mM DTT, 10 mM MgCl_2_) at 37 °C for 60 min. The reaction was quenched with 6× gel loading dye (NEB). The digested product was purified using Gel Extraction Kit (CWBiotech) following the manufacturer’s protocol and subjected to Sanger sequencing.

For 5’-FAM labeled ssDNA cleavage, 20 nM ssDNA was incubated with 500 nM IS607 TnpB RNP complex in cleavage buffer (25 mM Tris-HCl, pH 8.0, 50 mM NaCl, 3 mM DTT, 10 mM MgCl_2_) at 37 °C, then 10 μL aliquots were removed at time intervals (0 min, 0.5 min, 1 min, 2 min, 5 min, 10 min and 15 min), and stopped by adding 10 μL of 2× TBE-urea gel loading buffer. The cleavage products were denatured and analyzed by 20% TBE-urea-PAGE gel electrophoresis. The oligo sequences used in this study are provided in Supplementary information, Table S5.

### The plasmid and endogenous genomic DNA interference in bacteria

100 ng of the IS607 TnpB RNP complex expressing plasmid and 100 ng of the targeted plasmid were co-transformed into *E. coli* C43 (DE3) and cultured at 30 °C on LB plates containing 50 μg/mL spectinomycin and 100 μg/mL kanamycin overnight. Monoclones were isolated from the plate and cultured in 2 mL LB medium with spectinomycin and kanamycins containing 0.6 mM IPTG at 30 °C, 220 rpm for 12 h. *E. coli* culture samples were serially diluted (10×). 5 μL of the culture was added to the LB medium plate with spectinomycin and kanamycin and cultured at 30 °C for 24 h, followed by counting and analysis. For DNA cleavage of the endogenous genomic DNA of *E. coli* C43(DE3), the genome-targeting sequences were constructed into the IS607 TnpB RNP complex expressing plasmid. The target sequences used in this assay are provided in Supplementary information, Table S5.

### HEK293F cell culture and transfection

HEK293F cells (Invitrogen) were cultured in SMM 293T-II medium (Sino Biological Inc.) at 37 °C under 5% CO_2_ with agitation at 120 rpm. When the cell density reached 1 × 10^6^ cells per mL, the plasmid containing IS607 TnpB and reRNA were transfected into the cells. For 10 mL of the transfection system, 10 μg of plasmid was pre-mixed with 100 μg of 25-kDa linear polyethylenimines (Polysciences) in 1 mL fresh medium for 30 min before transfection, followed by dilution of the cell culture to 0.7 × 10^6^ cells per mL with fresh medium. Transfected cells were cultured at 37 °C under 5% CO_2_ with agitation at 120 rpm for 72 h before they were collected.

### NGS of HEK293F genomic DNA samples

After 72 h transfection, cells expressing green fluorescent protein were sorted out by flow cytometry (BD FACSAria Fusion). Genomic DNA was extracted using the Universal Genomic DNA Kit (CWBiotech) following the manufacturer’s protocol. The genomic region flanking the target site was amplified by PCR with GoTaq Green Master Mix (Promega). Equal amounts of PCR product were sent for NGS at the Hi-TOM platform.^71^ Indels were analyzed by the Hi-TOM platform (http://121.40.237.174/Hi-TOM/). The primers used in this study are provided in Supplementary information, Table S5.

### Cryo-EM data collection and image processing

For cryo-EM studies, 5 μL of purified protein was applied to glow-discharged holey Au 300 grids (Quantifoil R1.2/1.3) mounted in the chamber of a FEI Vitrobot Mark IV. The Vitrobot was operated at 4 °C, with a blot force of “0”, and a blotting time of 3–5 s, and 100% humidity. Grids were plunged into liquid ethane for cryo-freezing. Cryo-grids were first screened in a Talos Arctica operated at 200 kV (equipped with a FEI Falcon 3 camera). Data collection was performed with a FEI Titan Krios operated at 300 kV with a FEI Falcon 3 camera using the Thermo Fisher Scientific EPU software. The nominal magnification of 96,000× and the defocus range between –1.2 μm and –2.5 μm were used for data collection. For each image stack (32 frames), a total dose is about 40.0 electrons/Å^2^ at a calibrated pixel size 0.86 Å.

For the structure of ISFba1 TnpB–reRNA–dsDNA, a total of 9957 micrographs were obtained for data processing (Supplementary information, Fig. S4). Movie stacks were drift-corrected, electron-dose weighted and two-fold binned using MotionCor2.^72^ The contrast transfer function (CTF) parameters of drift-corrected micrographs were estimated by the program Gctf.^73^ Around 10,000 particles were manually picked to generate two-dimensional (2D) templates for automatic particle-picking in RELION3.1.^74^ With the templates, 6,783,728 particles were picked out and further used for 2D and 3D classifications, yielding a total of 369,379 particles for the 3D reconstruction. After the CTF refinement and Bayesian polishing, another round of 3D reconstruction was performed, yielding the final reconstruction of 3.0 Å. The resolution was evaluated based on the gold standard Fourier shell correlation (threshold = 0.143), and the local resolution map was estimated by ResMap.^75^

### Model building and structure refinement

For the structure of ISFba1 TnpB–reRNA–dsDNA, model building was carried out based on the 3.0 Å reconstruction map. The atomic coordinate of ISFba1 TnpB–reRNA–dsDNA was built de novo, and manual adjusted using COOT.^76^ The model was then refined using phenix.real_space_refine^77^ application with secondary structure and geometry restraints. The final models were evaluated by MolProbity^78^ and Ramachandran plot.^79^ Statistics of the map reconstruction and model refinement are presented in Supplementary information, Table S1. Structural figures were prepared with PyMOL^80^ and Chimera.^81^

### Genomic and metagenomic data collection

To perform a comprehensive analysis of microbiome, we downloaded 1520 non-redundant, high-quality draft genomes generated from fecal samples of healthy humans,^35^ 19,165 complete bacteria genome sequences from the NCBI FTP site (ftp://ftp.ncbi.nih.gov/genomes/Bacteria/),^29^ 26,097 bacteria genome sequences from IMG database (https://img.jgi.doe.gov/),^32^ 12,715 genomes recovered from marine metagenome^34^ and 204,938 assembled metagenome and isolated genome from human gastrointestinal curated by MGnify.^30^ Furthermore, we also downloaded 62,158, 145,115 and 96,709 unassembled metagenomic samples covering various biomes curated by GOLD,^33^ MGnify^30^ and HumanMetagenomeDB,^31^ respectively. We excluded duplicated samples, and finally a total of 297,982 samples were used for further analysis.

### Metagenomic data assembly

FASTQ files of 297,982 metagenomic sequencing data were extracted from SRA format using fastq-dump with the option “-split-3” (https://github.com/ncbi/sra-tools). The quality control was performed by KneadData^82^ with default parameters, including trimming overrepresented reads, adapters, tandem repeat using and filtering human contamination by comparing with hg19 human reference genome for UCSC.^83^ The clean data were assembled using MEGAHIT^84^ with default parameters, and we got 5,505,172 contigs longer than 1 kb across the 297,982 metagenomic samples. We excluded contigs with 100% redundancy using CD-HIT,^85^ and finally a total of 5,297,689 contigs were used for further analysis.

### Taxonomy annotation

Taxonomies of genomic and assembled metagenomic data were obtained from the NCBI microbial genomes^29^ and MGnify^30^ portal, and unassembled metagenomic data were annotated by a custom strategy. We identified proteins in the 5,297,689 contigs by Prodigal^86^ with the ‘-p meta’, which is an optimized parameter for metagenome. Each protein in the contigs was aligned to NCBI protein database by DIAMOND^87^ with identity > 40% and coverage > 70%, and the taxonomy of the best hit was transferred to each protein, the taxonomy of contig was assigned as the taxonomic rank having > 70% agreement across annotated proteins. Meanwhile, each contig was aligned to NCBI nucleotide database through BLASTN^43^ with *e* < 0.01 and the taxonomy of the best hit was transferred to each contig. If the taxonomy rank annotated by both methods is the same, the taxonomy is adopted. Otherwise, the taxonomy is not determined.

### Annotation for defense island

The defense island is a non-randomly contiguous string of genes flanked by housekeeping genes, at least one of which belongs to a known defense gene family.^6^ 3009 housekeeping-related and 2085 defense-related profiles^6,36,37,88^ were collected for building the housekeeping gene database (HKGD) and the defense gene database (DEGD) (Supplementary information, Table S1). Housekeeping-related genes were annotated using RPS-BLAST^43^ by comparing with HKGD with *e* value cutoff of 1E–2, and defense-related gene were annotated using Hmmscan^40^ by comparing with DEGD with *e* value cutoff of 1E–4. The final DI was annotated as the region containing defense-related genes flanked by housekeeping genes.

### Detection of the RNAs within IGRs

IGRs consist of a series of functional elements.^89^ We extracted the IGRs from the DI region, discarding all IGRs shorter than 15 nt, and predicting the potential RNAs with conserved secondary structure using INFERNAL^90^ based on Rfam^38^ database with default parameter.

### Clustering genes proximal to RNAs within IGRs

Ten proteins both upstream and downstream of predicted RNAs within IGRs were clustered by a two-step procedure. Firstly, all proteins were initially clustered by comparing to each other using MMseqs^91^ with *e* value cutoff of 1E–3, sensitivity of 7.5, identity of 0.3, and coverage of 0.6. Re-clustering was then performed to integrate clusters with remote homology. Proteins in each initial cluster were aligned using MAFFT^92^ and HMM profiles were constructed using HMMER^40^ based on the results of multiple sequence alignment. Subsequently, the similarity between each protein of the initial clustering and the HMM profile was quantified using Hmmscan^40^ with *e* value cutoff of 1E–5, and used as input weights for the Markov clustering algorithm (MCL)^93^ to form the final gene families with an inflation rate of 2.0.

### Predicted secondary structure for reRNA

Using results of “clustering genes proximal to RNAs within IGRs”, we found that the RAGATH-18 RNA stably co-existed with IS607 TnpB. All IGRs containing RAGATH-18 RNA were aligned using MAFFT^92^ and conserved regions were predicted for secondary structure using R-scape.^94^

### Clustering RAGATH-18 RNA-associated proteins

All RAGATH-18 RNA-associated proteins were collected, and the proteins with 90% redundancy were excluded using CD-HIT.^85^ The remaining proteins were clustered using MMseqs^91^ with *e* value cutoff of 1E–3, sensitivity of 7.5, identity of 0.3, and coverage of 0.6. Similarity inter or inner clusters was the median of similarity scores for all corresponding proteins, and the similarity score of any pair of proteins was defined as sequence identity multiplied by sequence coverage calculated using BLASTP.^43^ The phylogenetic trees were built based on the resulting alignments using FastTree2,^95^ taking the WAG model by 1000 resamples, rescaling the branch lengths, and computing a Gamma20-based likelihood. The screening of representative proteins was conducted through sequence similarity clustering, followed by filtering based on taxonomy, with up to 6 proteins per species. This process yielded 43 candidate proteins from a pool of 10,258 predicted IS607 TnpB systems of 9172 bacterial strains. Additionally, in the 293F genomic DNA editing assay, 14 TnpBs were identified with low homology (< 50% sequence similarity) to ISFba1 within the same species or high homology (> 80% sequence similarity) to ISFba1 in different species. All the 57 TnpBs were listed in Supplementary information, Table S2.

### Statistics

Statistical analyses and graphing were done in Prism (Graphpad). Two-tailed unpaired *t*-tests were used to compare the means of target and non-target DNA interference efficiencies in *E. coli* and 293F cells.

### Supplementary information

Supplementary information, Fig.S1

Supplementary information, Fig.S2

Supplementary information, Fig.S3

Supplementary information, Fig.S4

Supplementary information, Fig.S5

Supplementary information, Fig.S6

Supplementary information, Fig.S7

Supplementary information, Fig.S8

Supplementary information, Fig.S9

Supplementary information, Table S1

Supplementary information, Table S2

Supplementary information, Table S3

Supplementary information, Table S4

Supplementary information, Table S5

## Data Availability

The atomic coordinate of ISFba1 TnpB–reRNA–target DNA has been deposited in the Worldwide Protein Data Bank (wwPDB) with accession code 8IAZ. The corresponding map has been deposited in the Electron Microscopy Data Bank (EMDB) with accession code EMD-35323. All raw and processed sequencing data used for this study are available to download via http://www.microbiome-bigdata.com/project/IS607_TnpB/. All other data needed to support the conclusions in this manuscript can be found in the main text or supplementary information. Any additional information required to reanalyze the data reported in this paper is available from the lead contact upon request.
