# Author Correction: Discovery and structural mechanism of DNA endonucleases guided by RAGATH-18-derived RNAs

**DOI:** 10.1038/s41422-024-00999-0

**Published:** 2024-07-23

**Authors:** Kuan Ren, Fengxia Zhou, Fan Zhang, Mingyu Yin, Yuwei Zhu, Shouyu Wang, Yan Chen, Tengjin Huang, Zixuan Wu, Jiale He, Anqi Zhang, Changyou Guo, Zhiwei Huang

**Affiliations:** 1 HIT Center for Life Sciences, School of Life Science and Technology, Harbin Institute of Technology, Harbin, Heilongjiang China; 2 Westlake Center for Genome Editing, Westlake Laboratory of Life Sciences and Biomedicine, School of Life Sciences, Westlake University, Hangzhou, Zhejiang China; 3 New Cornerstone Science Laboratory, Shenzhen, Guangdong China

**Keywords:** Biological techniques, Cryoelectron microscopy

Correction to: *Cell Research* 10.1038/s41422-024-00952-1, published online 04 April 2024

In the version of this article initially published, we found one of the descriptions was given incorrectly in the section of “IS607 TnpB-mediated genome editing in human cells”. The sentence

“In contrast to SpCas9, ISFba1 TnpB tolerated more single mismatches between the guide and target sequences”

has been corrected as follows:

“In contrast to ISFba1 TnpB, SpCas9 tolerated more single mismatches between the guide and target sequences”. The authors apologize for this carelessness. The original article has been corrected.

