# Supplementary material for: Discovery and structural mechanism of DNA endonucleases guided by RAGATH-18-derived RNAs
Source: Cell Res. 2024 Apr 4;34(5):370–85. doi: 10.1038/s41422-024-00952-1 (PMC11061315; doi:10.1038/s41422-024-00952-1)
Supplement: Supplementary file 1 — Supplementary information, Fig.S1 [file 41422_2024_952_MOESM1_ESM.pdf]

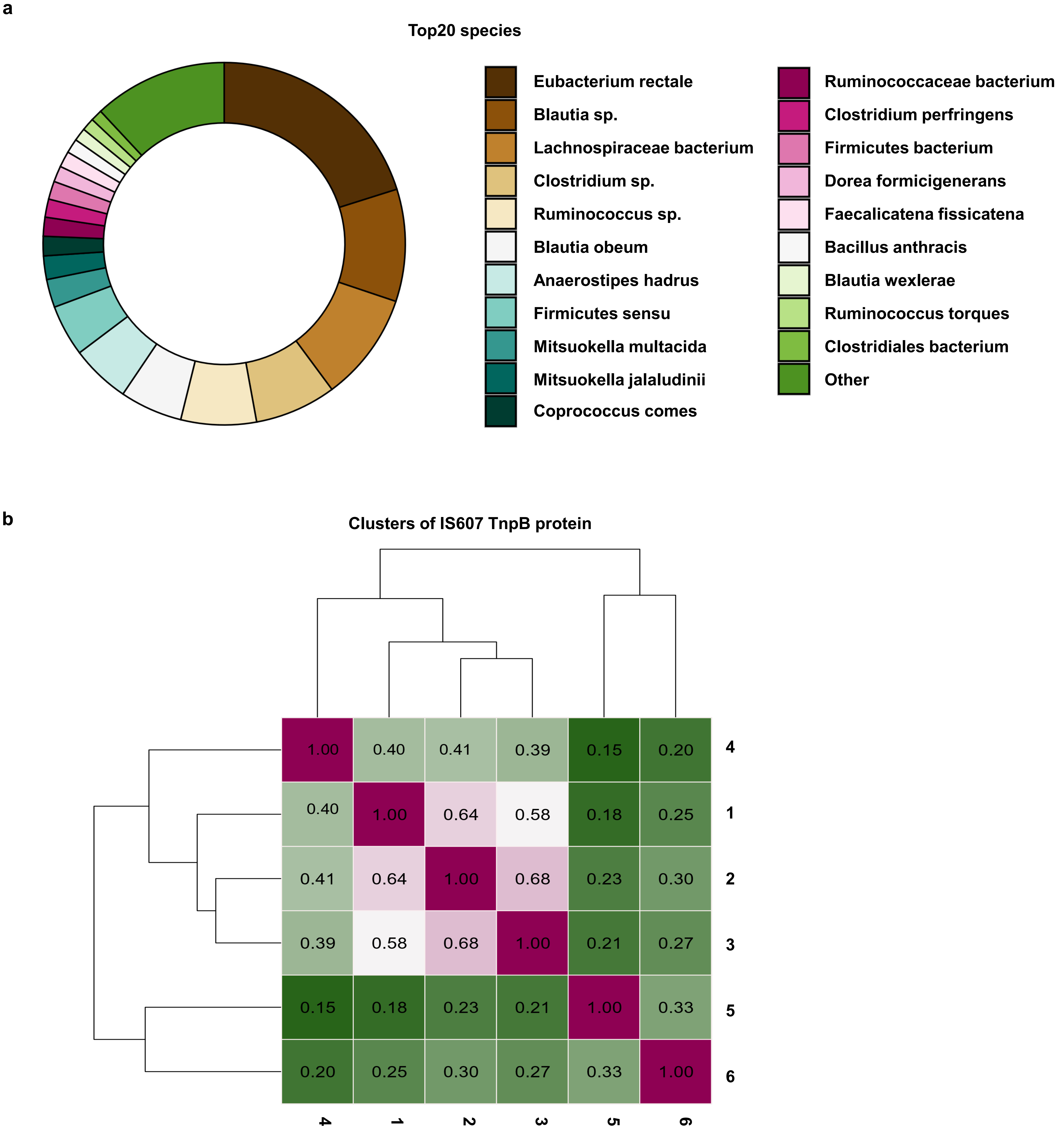


**Supplementary information, Fig.S1: Characterization of RAGATH-18 RNA and its adjacent protein.**
**a** The taxonomy of RAGATH-18 RNA-associated protein at species rank, only the top 20 taxonomies are shown.
**b** Heatmap displays the similarity between six clusters of RAGATH-18 RNA-associated protein. The similarity score shown in cells is defined as the product of sequence identity and coverage.
