# Supplementary material for: Discovery and structural mechanism of DNA endonucleases guided by RAGATH-18-derived RNAs
Source: Cell Res. 2024 Apr 4;34(5):370–85. doi: 10.1038/s41422-024-00952-1 (PMC11061315; doi:10.1038/s41422-024-00952-1)
Supplement: Supplementary file 2 — Supplementary information, Fig.S2 [file 41422_2024_952_MOESM2_ESM.pdf]

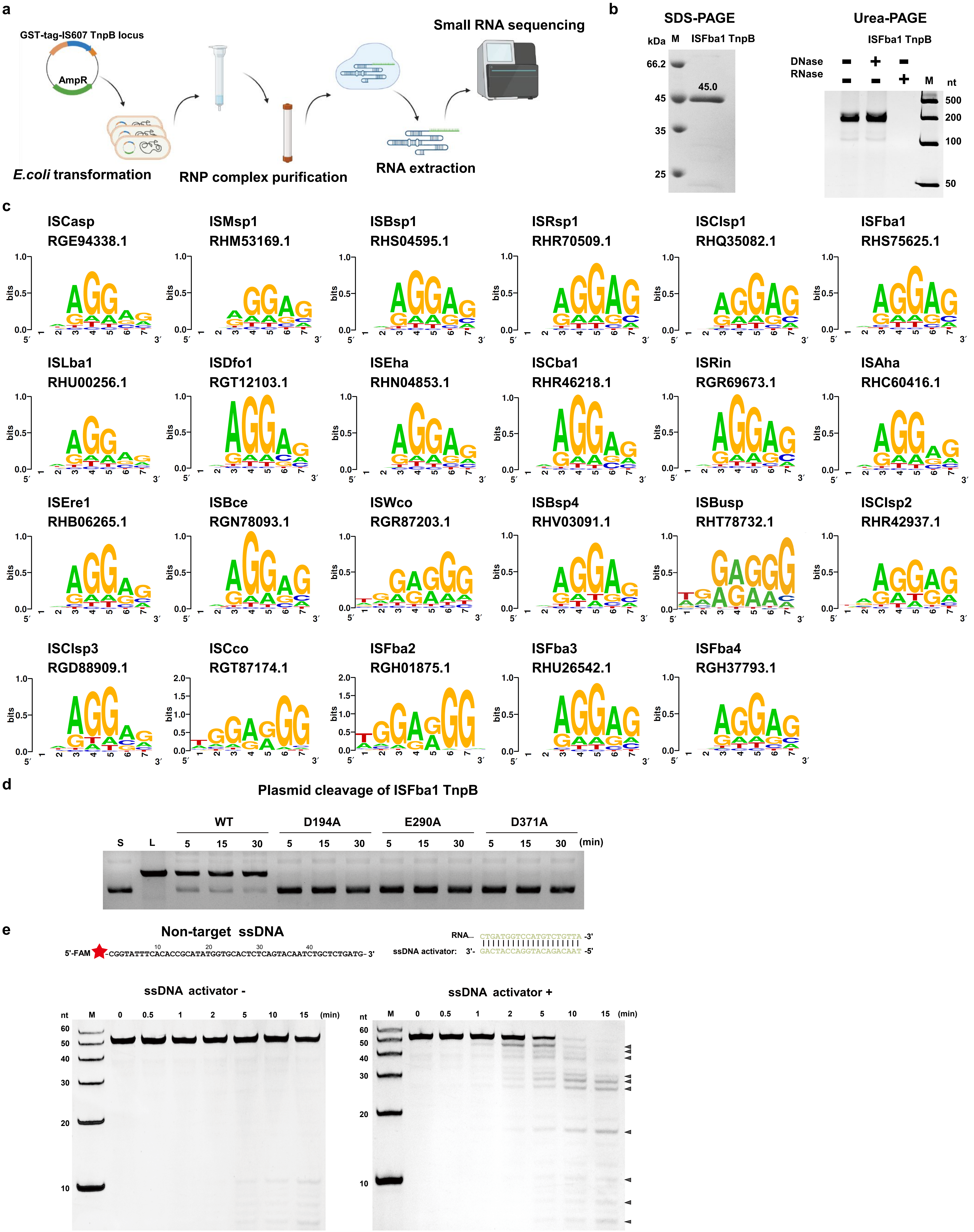
a
GST-tag-IS607 TnpB locus
AmpR
E.coli transformation
RNP complex purification
RNA extraction
Small RNA sequencing
b
SDS-PAGE
kDa M ISFba1 TnpB
66.2
45
35
25
45.0
Urea-PAGE
ISFba1 TnpB
DNase
RNase
M
nt
500
200
100
50
c
ISCasp RGE94338.1
ISMsp1 RHM53169.1
ISBsp1 RHS04595.1
ISRsp1 RHR70509.1
ISClsp1 RHQ35082.1
ISFba1 RHS75625.1
ISLba1 RHU00256.1
ISDfo1 RGT12103.1
ISEha RHN04853.1
ISCba1 RHR46218.1
ISRin RGR69673.1
ISAha RHC60416.1
ISEre1 RHB06265.1
ISBce RGN78093.1
ISWco RGR87203.1
ISBsp4 RHV03091.1
ISBusp RHT78732.1
ISClsp2 RHR42937.1
ISClsp3 RGD88909.1
ISCco RGT87174.1
ISFba2 RGH01875.1
ISFba3 RHU26542.1
ISFba4 RGH37793.1
bits
d
Plasmid cleavage of ISFba1 TnpB
S L
WT
D194A
E290A
D371A
5 15 30 5 15 30 5 15 30 5 15 30 (min)
e
Non-target ssDNA
5'-FAM -CGGTATTTCACACCGCATATGGTGCACTCTCAGTACAATCTGCTCTGATG- 3'
RNA... CTGATGGTCCATGTCTGTTA -3'
ssDNA activator: 3'- GACTACCAGGTACAGACAAT -5'
ssDNA activator -
ssDNA activator +
nt M 0 0.5 1 2 5 10 15 (min)
60
50
40
30
20
10

Supplementary information, Fig.S2: Characterization of the DNA cleavage activity of IS607 TnpB systems.
a Schematic of small RNA sequencing of the IS607 TnpB RNP complex.
b The SDS-PAGE(left) and 10% TBE-urea-PAGE(right) of the purified ISFba1 RNA-protein complex.
c Weblogo of TAM sequences for 23 IS607 TnpB systems from 22 bacterial strains of 17 different species. The detailed information of the different IS607 TnpB proteins is provided in Supplementary information, Table S2.
d In vitro time gradient plasmid cleavage mediated by wild type ISFba1 TnpB and mutants (D194A, E290A, D371A). The agarose gel was resolved by EB staining. S, supercoil plasmid. L, linearized plasmid. Data shown are representative of three independent experiments.
e ISFba1 TnpB-mediated 5'-FAM labeled ssDNA cleavage with a 20-nt target ssDNA activator monitored by 20% TBE-urea-PAGE gel electrophoresis. Cleavage products are labeled by triangles. M, ssDNA marker.
ssDNA activator-, reaction without ssDNA activator. ssDNA activator+, reaction with ssDNA activator. Data shown are representative of three independent experiments.
