# Supplementary material for: Discovery and structural mechanism of DNA endonucleases guided by RAGATH-18-derived RNAs
Source: Cell Res. 2024 Apr 4;34(5):370–85. doi: 10.1038/s41422-024-00952-1 (PMC11061315; doi:10.1038/s41422-024-00952-1)
Supplement: Supplementary file 3 — Supplementary information, Fig.S3 [file 41422_2024_952_MOESM3_ESM.pdf]

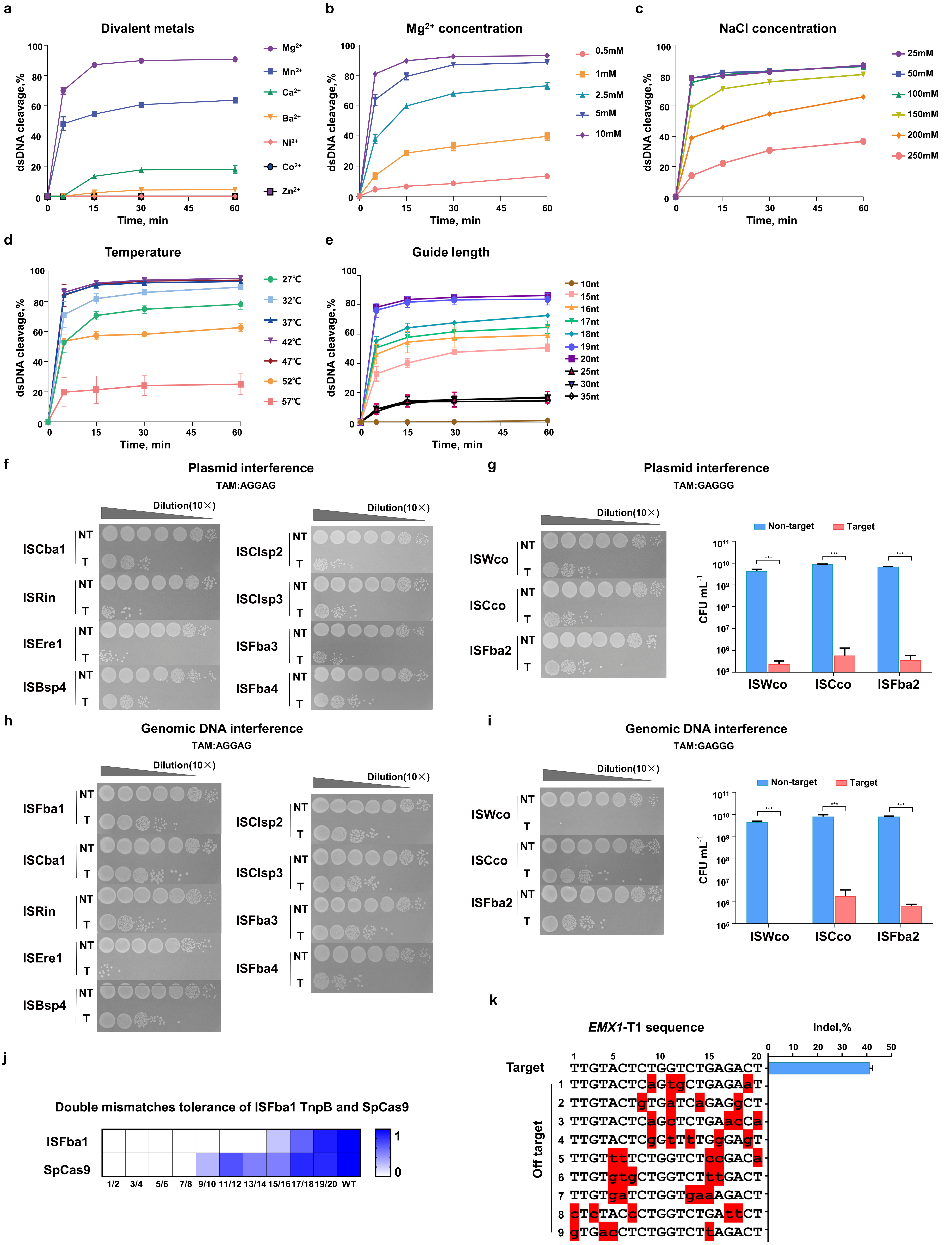
a
Divalent metals
dsDNA cleavage,%
Time, min
Mg2+
Mn2+
Ca2+
Ba2+
Ni2+
Co2+
Zn2+
b
Mg2+ concentration
0.5mM
1mM
2.5mM
5mM
10mM
c
NaCl concentration
25mM
50mM
100mM
150mM
200mM
250mM
d
Temperature
27℃
32℃
37℃
42℃
47℃
52℃
57℃
e
Guide length
10nt
15nt
16nt
17nt
18nt
19nt
20nt
25nt
30nt
35nt
f
Plasmid interference
TAM:AGGAG
Dilution(10×)
NT
T
ISCba1
ISRin
ISEre1
ISBsp4
ISClsp2
ISClsp3
ISFba3
ISFba4
g
Plasmid interference
TAM:GAGGG
ISWco
ISCco
ISFba2
Non-target
Target
CFU mL-1
h
Genomic DNA interference
TAM:AGGAG
ISFba1
ISCba1
ISRin
ISEre1
ISBsp4
ISClsp2
ISClsp3
ISFba3
ISFba4
i
Genomic DNA interference
TAM:GAGGG
ISWco
ISCco
ISFba2
j
Double mismatches tolerance of ISFba1 TnpB and SpCas9
ISFba1
SpCas9
1/2 3/4 5/6 7/8 9/10 11/12 13/14 15/16 17/18 19/20 WT
k
EMX1-T1 sequence
Indel,%
Target TTGTACTCTGGTCTGAGACT
Off target
1 TTGTACTCaGtgCTGAGAaT
2 TTGTACTgTGaTCaGAGgCT
3 TTGTACTCaGcTCTGAacCa
4 TTGTACTCgGtTtTGgGAgT
5 TTGTttTCTGGTCTccGACa
6 TTGTgtgCTGGTCTttGACT
7 TTGTgaTCTGGTgaaAGACT
8 cTcTACcCTGGTCTGAttCT
9 gTGacCTCTGGTCTtAGACT

Supplementary information, Fig.S3: Biochemical features of ISFba1 TnpB-mediated cleavage, DNA interference activity of IS607 TnpB systems in *E. coli* and the specificity of ISFba1 TnpB in 293F.

Tests of different reaction conditions on the dsDNA cleavage activity of ISFba1 TnpB.

a Divalent metal types. **b** Mg^{2+} concentrations. **c** NaCl concentrations. **d** Temperatures. **e** Guide length. Data represent mean ± SD of three biological replicates.

Culture samples of IS607 TnpB-mediated plasmid interference using **f** 5'-AGGAG TAM and **g** 5'-GAGGG TAM (left panel) in *E. coli* were serially diluted (10×) and selected by the media supplemented with Kan and Spe.

g Quantification of the plasmid interference assay mediated by ISWco, ISCco and ISFba2 TnpB using 5'-GAGGG TAM (right panel). Data represent mean ± SD of three biological replicates. Two-tailed unpaired t-test: ***P < 0.001.

The culture samples of genomic DNA interference assay mediated by IS607 TnpB family proteins using **h** 5'-AGGAG TAM and **i,** 5'-GAGGG TAM (left panel) in *E. coli* were serially diluted (10×) and selected.

i Quantification of the genomic DNA interference assay mediated by ISWco, ISCco and ISFba2 TnpB using 5'-GAGGG TAM (right panel). NT, non-target control group. T, target treated group. Data represent mean ± SD of three biological replicates. Two-tailed unpaired t-test: ***P < 0.001.

j Heatmap of the double mismatche tolerance of ISFba1 and SpCas9 on the *EMX1* target sites. 1-20, mismatch position 1-20. WT, original guide sequence. Data shown are representative of three independent experiments.

k ISFba1 TnpB-mediated indel frequencies at predicted genomic off-target loci. Mismatched bases were highlighted by the red squares. Data shown are representative of three independent experiments. The guide sequences are provided in Supplementary information, Table S5.
