# Supplementary material for: Discovery and structural mechanism of DNA endonucleases guided by RAGATH-18-derived RNAs
Source: Cell Res. 2024 Apr 4;34(5):370–85. doi: 10.1038/s41422-024-00952-1 (PMC11061315; doi:10.1038/s41422-024-00952-1)
Supplement: Supplementary file 4 — Supplementary information, Fig.S4 [file 41422_2024_952_MOESM4_ESM.pdf]

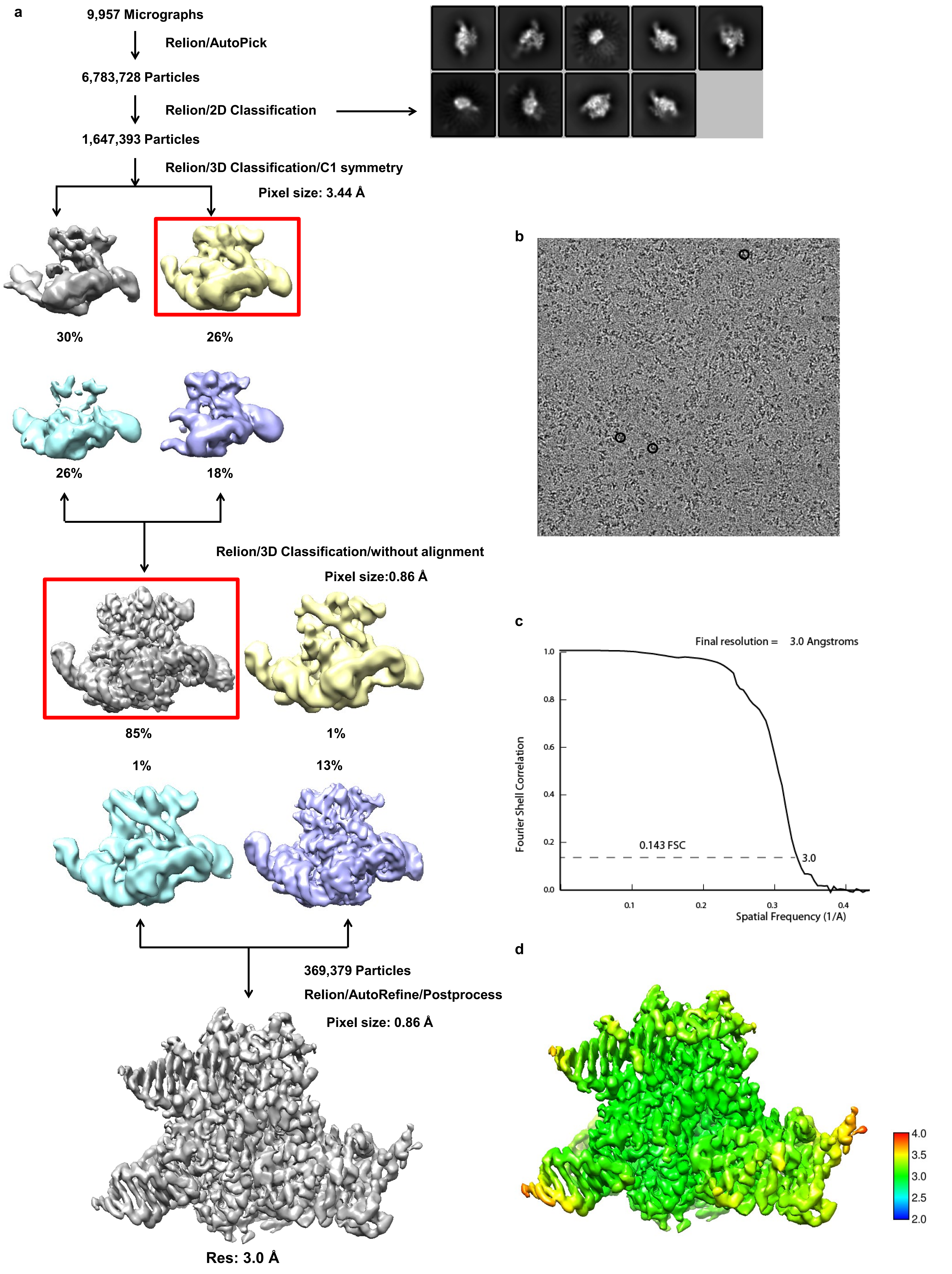

a
9,957 Micrographs
Relion/AutoPick
6,783,728 Particles
Relion/2D Classification
1,647,393 Particles
Relion/3D Classification/C1 symmetry
Pixel size: 3.44 Å
30%
26%
26%
18%
Relion/3D Classification/without alignment
Pixel size:0.86 Å
85%
1%
1%
13%
369,379 Particles
Relion/AutoRefine/Postprocess
Pixel size: 0.86 Å
Res: 3.0 Å
b
c
Final resolution = 3.0 Angstroms
Fourier Shell Correlation
0.143 FSC
3.0
Spatial Frequency (1/A)
d

Supplementary information, Fig.S4: Cryo-EM image processing procedure of the ISFba1 TnpB-reRNA-DNA complex.

a Image processing workflow of the ISFba1 TnpB-reRNA-DNA complex.
b A representative raw cryo-EM image of the ISFba1 TnpB-reRNA-DNA complex. The particles that were picked for classification are labeled with circles.
c Gold standard FSC plot for the final 3D reconstruction of the ISFba1 TnpB-reRNA-DNA complex.
d Resolution map for the final 3D reconstruction of the ISFba1 TnpB-reRNA-DNA complex. The color code for resolutions, shown with the unit Å, is calculated using cryoSPARC.
