# Supplementary material for: Discovery and structural mechanism of DNA endonucleases guided by RAGATH-18-derived RNAs
Source: Cell Res. 2024 Apr 4;34(5):370–85. doi: 10.1038/s41422-024-00952-1 (PMC11061315; doi:10.1038/s41422-024-00952-1)
Supplement: Supplementary file 5 — Supplementary information, Fig.S5 [file 41422_2024_952_MOESM5_ESM.pdf]

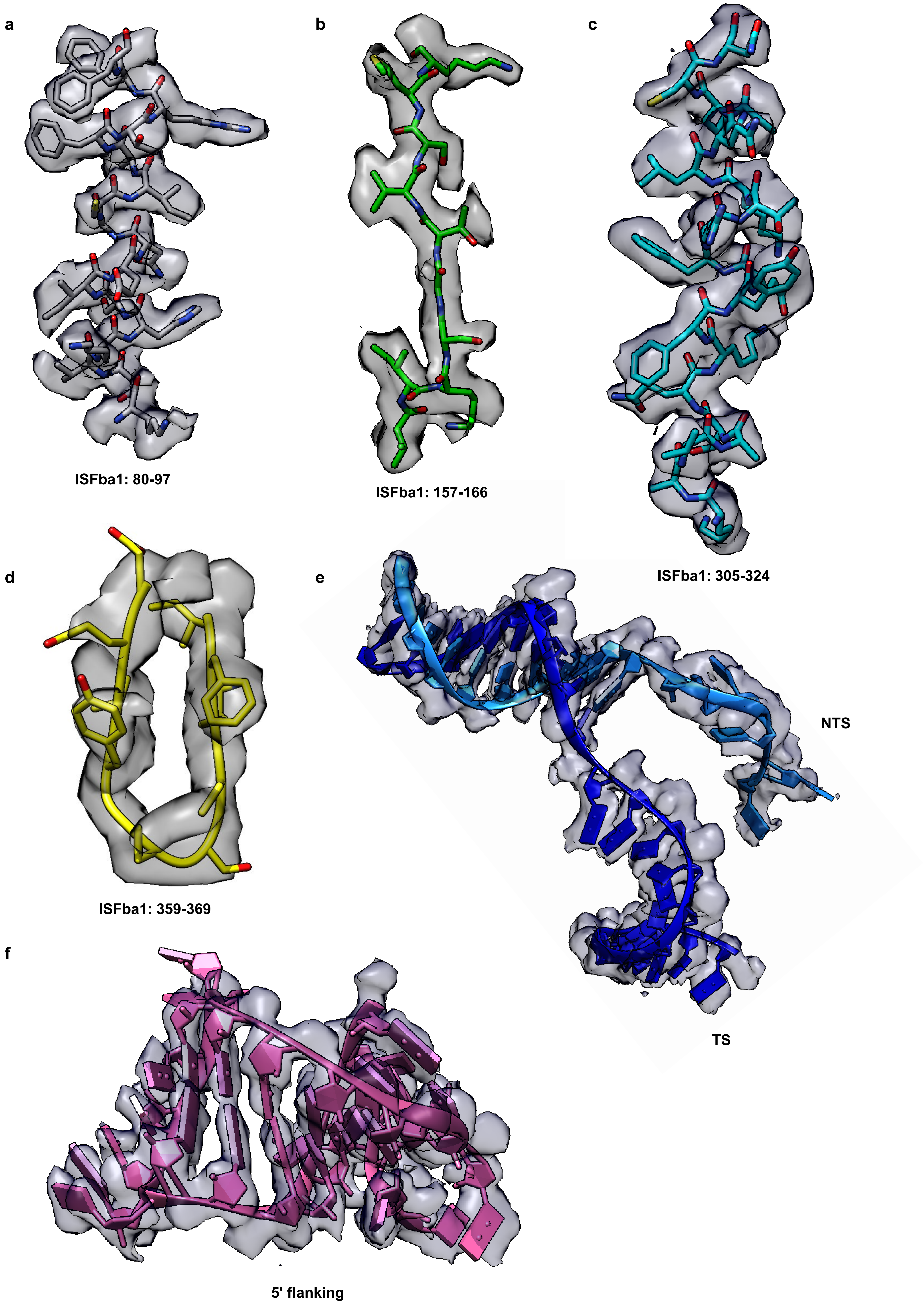

Supplementary information, Fig.S5: Representative local map density for the IS607 TnpB complex.
a-d Cryo-EM densities for representative IS607 TnpB protein regions.
e Cryo-EM densities for the target and non-target DNA strands.
f Cryo-EM densities for representative reRNA regions.
