# Supplementary material for: Discovery and structural mechanism of DNA endonucleases guided by RAGATH-18-derived RNAs
Source: Cell Res. 2024 Apr 4;34(5):370–85. doi: 10.1038/s41422-024-00952-1 (PMC11061315; doi:10.1038/s41422-024-00952-1)
Supplement: Supplementary file 6 — Supplementary information, Fig.S6 [file 41422_2024_952_MOESM6_ESM.pdf]

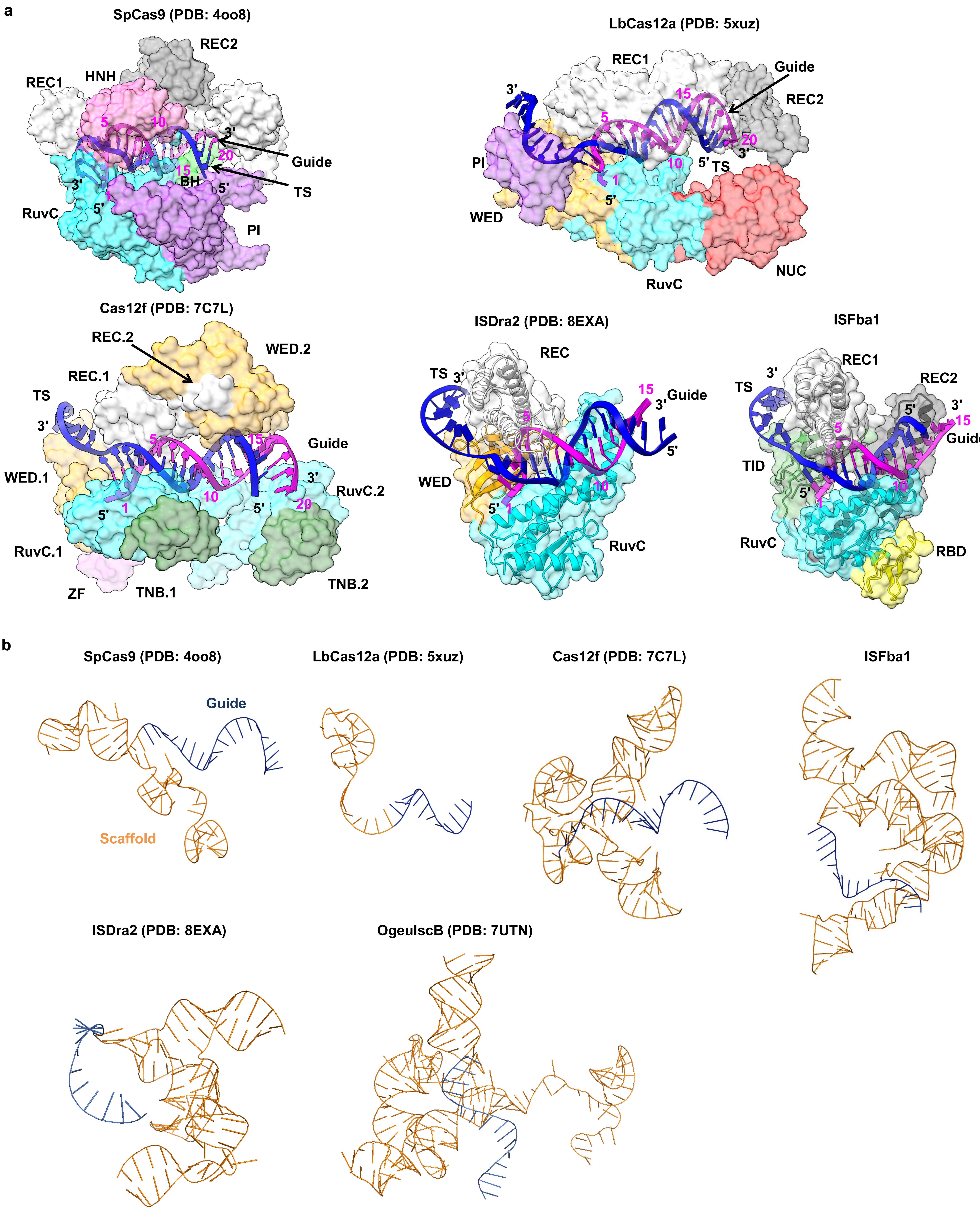

Supplementary information, Fig.S6: Structural comparison of SpCas9, LbCas12a, Cas12f, ISDra2 TnpB, OgeuIscB and ISFba1 TnpB.

a The RNA-DNA heteroduplex recognition by the SpCas9, LbCas12a, Cas12f, ISDra2 and ISFba1 TnpB.

b Structural comparison between guide and scaffold RNAs of the SpCas9, LbCas12a, Cas12f, ISDra2, OgeuIscB and ISFba1 TnpB.
