# Supplementary material for: Discovery and structural mechanism of DNA endonucleases guided by RAGATH-18-derived RNAs
Source: Cell Res. 2024 Apr 4;34(5):370–85. doi: 10.1038/s41422-024-00952-1 (PMC11061315; doi:10.1038/s41422-024-00952-1)
Supplement: Supplementary file 7 — Supplementary information, Fig.S7 [file 41422_2024_952_MOESM7_ESM.pdf]

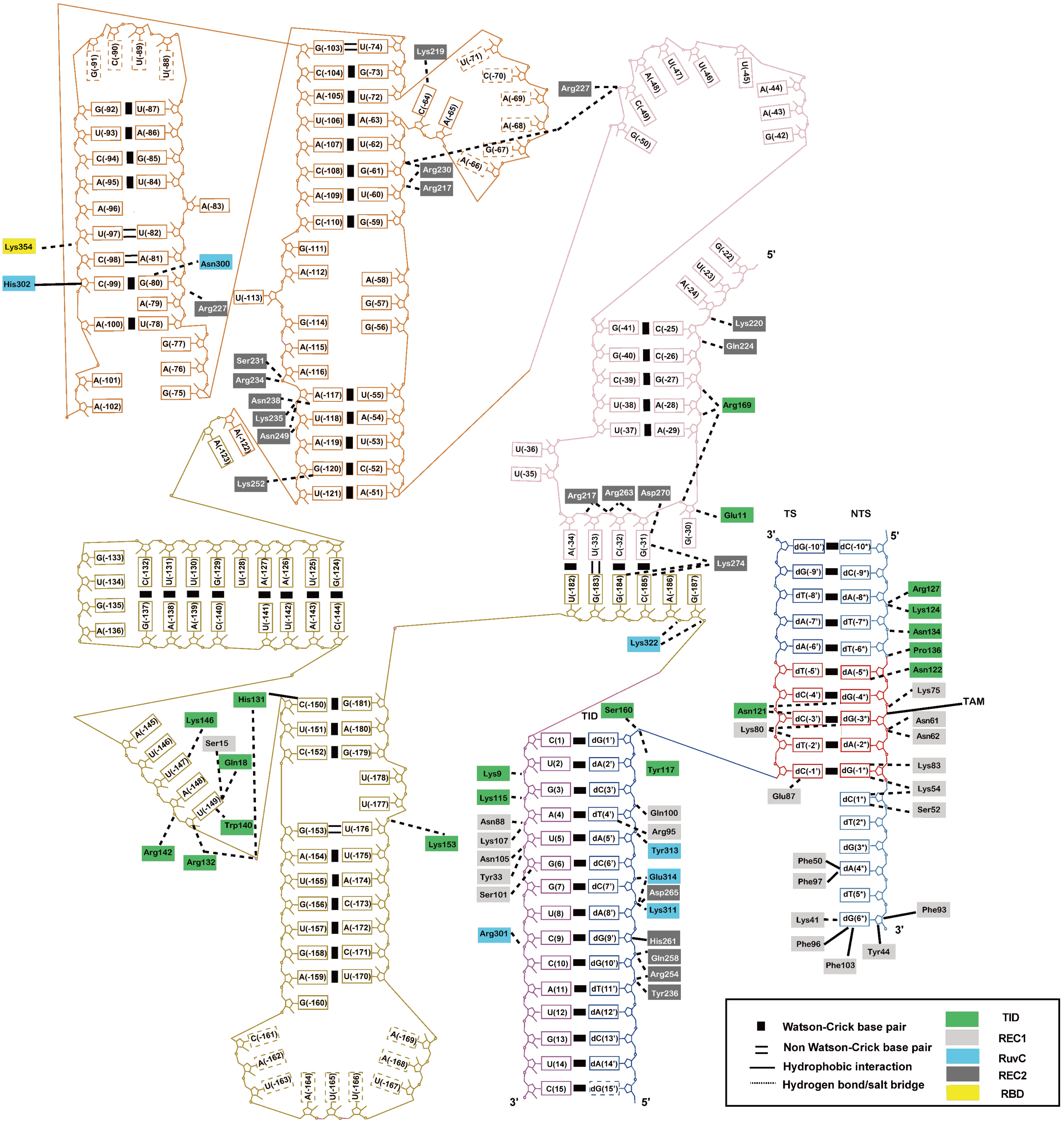

Supplementary information, Fig.S7: Diagram of nucleic acid recognition by ISFba1 TnpB.
Schematic of reRNA, NTS and TS strands recognition in the ISFba1 TnpB ternary complex. Domains and residues are colored according to Fig.6. Hydrogen bonds and salt bridges are labeled as dashed lines. Stackings and hydrophobic interactions are labeled as solid lines.
