# Supplementary material for: Discovery and structural mechanism of DNA endonucleases guided by RAGATH-18-derived RNAs
Source: Cell Res. 2024 Apr 4;34(5):370–85. doi: 10.1038/s41422-024-00952-1 (PMC11061315; doi:10.1038/s41422-024-00952-1)
Supplement: Supplementary file 9 — Supplementary information, Fig.S9 [file 41422_2024_952_MOESM9_ESM.pdf]

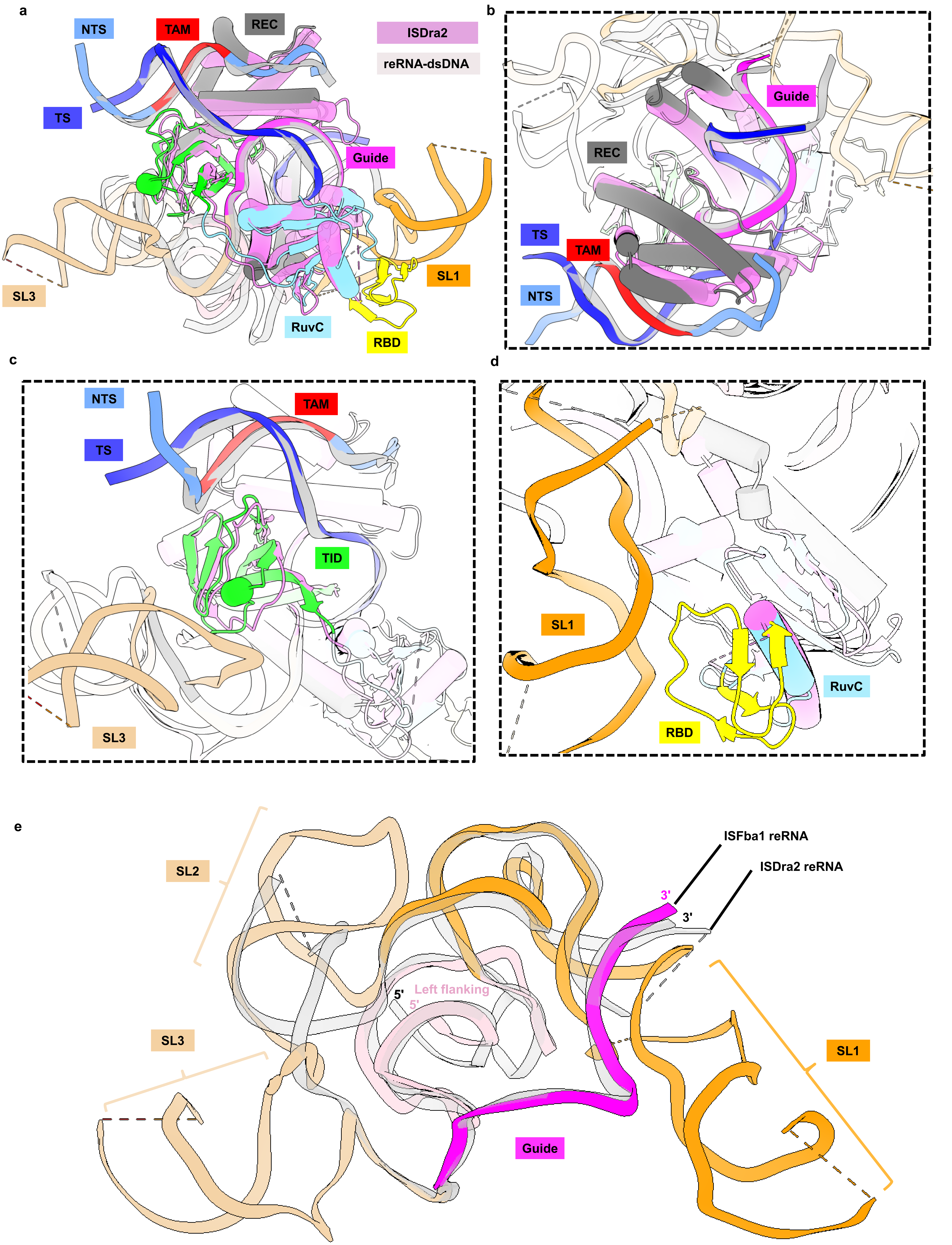
a
NTS
TAM
REC
ISDra2
reRNA-dsDNA
TS
Guide
SL1
SL3
RuvC
RBD
b
Guide
REC
TS
TAM
NTS
c
NTS
TAM
TS
TID
SL3
d
SL1
RuvC
RBD
e
ISFba1 reRNA
ISDra2 reRNA
SL2
3'
3'
5'
Left flanking
5'
SL3
SL1
Guide

Supplementary information, Fig.S9: Structural Comparison of ISFba1 with ISDra2 TnpB.
a Structural alignment of ISFba1 TnpB-reRNA-dsDNA and ISDra2-reRNA-dsDNA (PDB ID:8EXA). The ISFba1-reRNA-dsDNA complex is colored as Fig. 5C. The ISDra2 and reRNA-dsDNA hybrid are colored violet and gray, respectively.
b-e Enlarged views are shown to highlight the conformational distinctions. The structural differences between ISFba1 TnpB-reRNA-dsDNA and ISDra2-reRNA-dsDNA are highlighted.
