# Supplementary material for: Discovery and structural mechanism of DNA endonucleases guided by RAGATH-18-derived RNAs
Source: Cell Res. 2024 Apr 4;34(5):370–85. doi: 10.1038/s41422-024-00952-1 (PMC11061315; doi:10.1038/s41422-024-00952-1)
Supplement: Supplementary file 12 — Supplementary information, Table S3 [file 41422_2024_952_MOESM12_ESM.pdf]

Supplementary information, Table S3: Cryo-EM data collection, refinement and validation statistics.

	ISFba1-reRNA-dsDNA
**Data collection and processing**	
Magnification	96,000
Voltage (kV)	300
Electron exposure ($e^{-}/Å^2$)	40.0
Defocus range (μm)	-1.2~-2.5
Pixel size (Å)	0.86
Symmetry imposed	C1
Initial particle images (no.)	6,783,728
Final particle images (no.)	369,379
Map resolution (Å)	3.0
FSC threshold	0.143
Map resolution range (Å)	4.0-3.0
**Refinement**	
Initial model used (PDB code)	N/A
Model resolution (Å)	3.0
FSC threshold	(0.143)
Model resolution range (Å)	4.0-3.0
Map sharpening *B* factor ($Å^2$)	-133.3
Model composition	
Nonhydrogen atoms	7396
Protein residues	384
Nucleotide	203
Ligand	1
B factors ($Å^2$)	
Protein	13.15
Nucleotide	27.74
Ligand	64.68
R.m.s. deviations	
Bond lengths (Å)	0.006
Bond angles (°)	0.794
**Validation**	
MolProbity score	2.40
Clashscore	8.37
Poor rotamers (%)	4.37
Ramachandran plot	
Favored (%)	92.67
Allowed (%)	7.07
Disallowed (%)	0.26
