# Supplementary material for: Discovery and structural mechanism of DNA endonucleases guided by RAGATH-18-derived RNAs
Source: Cell Res. 2024 Apr 4;34(5):370–85. doi: 10.1038/s41422-024-00952-1 (PMC11061315; doi:10.1038/s41422-024-00952-1)
Supplement: Supplementary file 8 — Supplementary information, Fig.S8 [file 41422_2024_952_MOESM8_ESM.pdf]

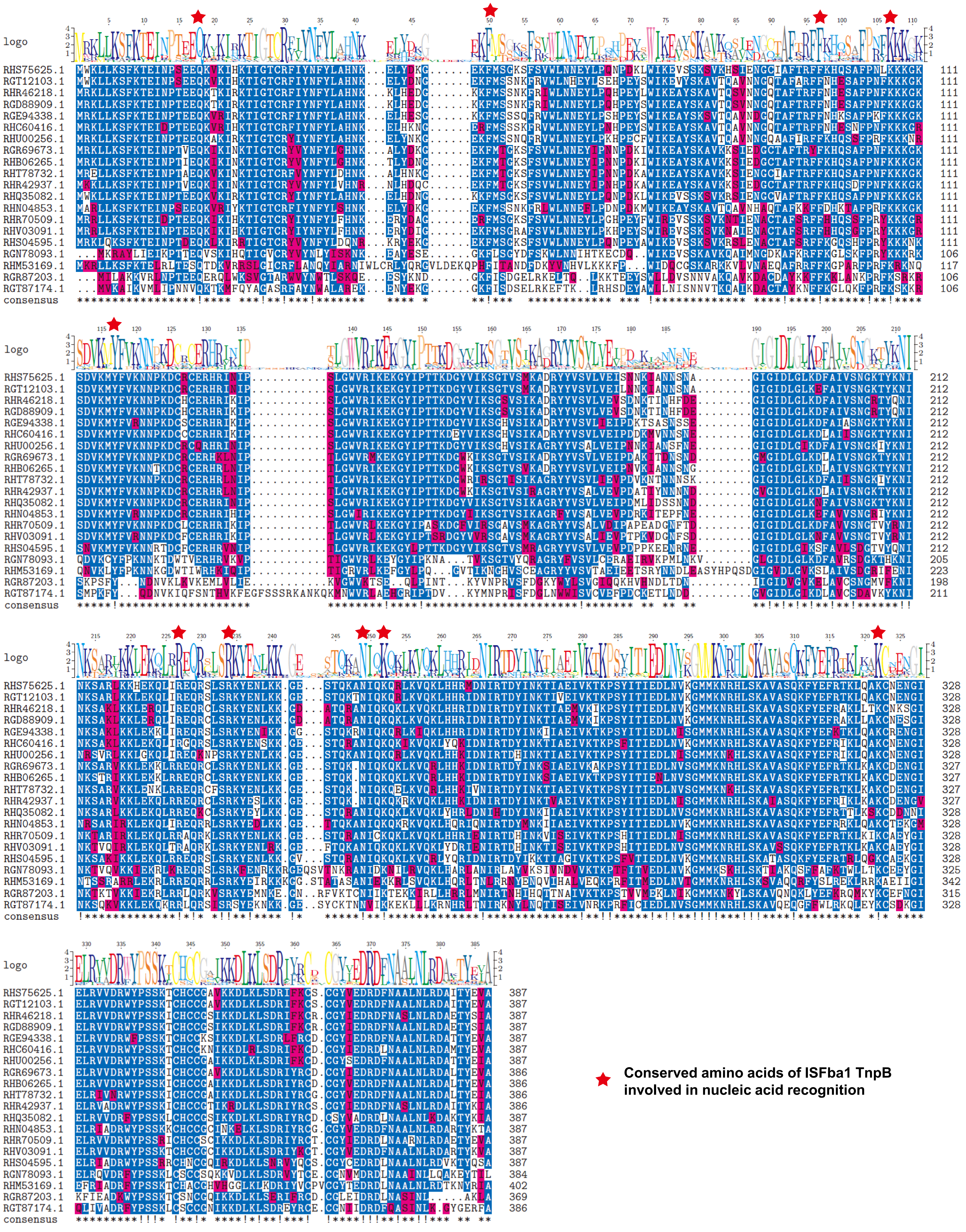

Supplementary information, Fig.S8: Protein sequence alignment of IS607 TnpB members with DNase activity. 20 IS607 TnpB proteins with DNase activity were aligned and analyzed. The conserved amino acids of ISFba1 TnpB involved in nucleic acid recognition are labeled by stars.
